# Benchmark single-step ethylene purification from ternary mixtures by a customized fluorinated anion-embedded MOF

**DOI:** 10.1038/s41467-023-35984-5

**Published:** 2023-01-25

**Authors:** Yunjia Jiang,, Yongqi Hu,, Binquan Luan,, Lingyao Wang,, Rajamani Krishna,, Haofei Ni,, Xin Hu, Yuanbin Zhang

**Affiliations:** 1 Key Laboratory of the Ministry of Education for Advanced Catalysis Materials, College of Chemistry and Life Sciences, Zhejiang Normal University, Jinhua, 321004 China; 2 IBM Thomas J. Watson Research, Yorktown Heights, New York, NY 10598 USA; 3 Van’t Hoff Institute for Molecular Sciences, University of Amsterdam, Science Park 904, 1098 XH Amsterdam, the Netherlands

**Keywords:** Chemical engineering, Carbon capture and storage

## Abstract

Ethylene (C_2_H_4_) purification from multi-component mixtures by physical adsorption is a great challenge in the chemical industry. Herein, we report a GeF_6_^2-^ anion embedded MOF (ZNU-6) with customized pore structure and pore chemistry for benchmark one-step C_2_H_4_ recovery from C_2_H_2_ and CO_2_. ZNU-6 exhibits significantly high C_2_H_2_ (1.53 mmol/g) and CO_2_ (1.46 mmol/g) capacity at 0.01 bar. Record high C_2_H_4_ productivity is achieved from C_2_H_2_/CO_2_/C_2_H_4_ mixtures in a single adsorption process under various conditions. The separation performance is retained over multiple cycles and under humid conditions. The potential gas binding sites are investigated by density functional theory (DFT) calculations, which suggest that C_2_H_2_ and CO_2_ are preferably adsorbed in the interlaced narrow channel with high aff0inity. In-situ single crystal structures with the dose of C_2_H_2_, CO_2_ or C_2_H_4_ further reveal the realistic host-guest interactions. Notably, rare C_2_H_2_ clusters are formed in the narrow channel while two distinct CO_2_ adsorption locations are observed in the narrow channel and the large cavity with a ratio of 1:2, which accurately account for the distinct adsorption heat curves.

## Abstract (web summary)

Ethylene (C_2_H_4_) purification from multi-component mixtures by physical adsorption is a great challenge in the chemical industry. Here authors present a GeF6^2-^ anion embedded MOF ZNU-6 with customized pore structure and pore chemistry for benchmark one-step C_2_H_4_ recovery from C_2_H_2_ and CO_2_ with record C_2_H_4_ productivity.

## Introduction

Ethylene (C_2_H_4_) is the foremost olefin as well as the highest volume product in the petrochemical industry, with an annual production capacity exceeding 214 million tons in 2021^1^. The manufacture of C_2_H_4_ and C_3_H_6_ accounts for 0.3% of global energy^2^. Current C_2_H_4_ production mainly relies on stream cracking of hydrocarbons^3–6^. Alternatively, oxidative coupling of methane (CH_4_) has emerged as a promising technique to produce C_2_H_4_, among which acetylene (C_2_H_2_) and carbon dioxide (CO_2_) are generated as byproducts and need to be deeply removed to produce polymer grade (>99.996%) C_2_H_4_^7^. Presently, multi-step purification process is adopted for purification of C_2_H_4_ from C_2_H_4_/C_2_H_2_/CO_2_ mixtures in industry^8^. C_2_H_2_ is removed by catalytic hydrogenation using expensive noble-metal catalysts or solvent extraction, which is either energy intensive or associated with pollution^9,10^. CO_2_ is removed by chemical adsorption using caustic soda, which causes huge waste of costly solvents^11^.

Physical adsorption offers potential to significantly reduce the energy footprint of separation processes^12–21^. Nonetheless, C_2_H_4_ purification from ternary C_2_H_4_/C_2_H_2_/CO_2_ mixtures remains an unmet challenge due to the similarity in molecular size and polarity (Supplementary Table 2), although separation of C_2_H_2_/C_2_H_4_^22–26^ or C_2_H_2_/CO_2_^27–32^ binary mixtures has been realized by a plethora of porous materials. Besides, single-step purification of C_2_H_4_ from ternary C_2_H_2_/C_2_H_4_/C_2_H_6_^33,34^ or quaternary C_2_H_2_/C_2_H_4_/C_2_H_6_/CO_2_^35^ mixtures has also been realized by several porous materials. To date, less than ten materials have been demonstrated to separate C_2_H_4_ from C_2_H_4_/C_2_H_2_/CO_2_, including activated carbons, zeolites, covalent organic frameworks and metal organic framework (MOFs)^36–39^. TIFSIX-17-Ni^36^, NTU-65^37^, and NTU-67^38^ are so far the three optimal materials. TIFSIX-17-Ni^36^ exhibits high C_2_H_2_/C_2_H_4_ and CO_2_/C_2_H_4_ selectivity due to the negligible uptake of C_2_H_4_ under ambient condition. However, the capacity of C_2_H_2_ (3.30 mmol/g) and CO_2_ (2.20 mmol/g) is relatively low due to the over-contracted channel. NTU-65^37^ can selectively capture C_2_H_2_ and CO_2_ by tuning the gate opening. However, the applied temperature must be at 263 K because lower temperatures lead to the adsorption of all the gases while higher temperatures cause the exclusion of CO_2_. NTU-67^38^ displays similar C_2_H_2_ (3.29 mmol/g) and CO_2_ (2.04 mmol/g) capacity, but the C_2_H_2_/C_2_H_4_ and CO_2_/C_2_H_4_ selectivity is greatly reduced as the C_2_H_4_ capacity (1.41 mmol/g) is relatively high. Additionally, the separation performance is deteriorated under humid conditions. Therefore, there is still a lack of ideal and stable materials to realize the simultaneous removal of C_2_H_2_ and CO_2_ in C_2_H_2_/CO_2_/C_2_H_4_ mixtures.

In this work, we reported a GeF_6_^2−^ anion embedded MOF ZNU-6 (ZNU = Zhejiang Normal University) with large cages (~8.5 Å diameter) connected by narrow interlaced channels (~4 Å diameter) for benchmark one-step C_2_H_4_ recovery from C_2_H_2_ and CO_2_. ZNU-6 is constructed by CuGeF_6_ and tri(pyridin-4-yl)amine (TPA) and exhibits excellent chemical stability. Static gas adsorption isotherms showed that ZNU-6 takes up 1.53/8.06 mmol/g of C_2_H_2_ and 1.46/4.76 mmol/g of CO_2_ at 0.01 and 1.0 bar (298 K), respectively. The calculated IAST selectivities for C_2_H_2_/C_2_H_4_ (1/99) and CO_2_/C_2_H_4_ (1/99) are 43.8–14.3 and 52.6–7.8 (0.0001–1.0 bar), respectively. The calculated *Q*_st_ values at near-zero loading for C_2_H_2_ and CO_2_ are 37.2 and 37.1 kJ/mol, indicative of its facility for material regeneration but much higher than that of C_2_H_4_ (29.0 kJ/mol). Modeling study indicates that there are two potential binding sites for C_2_H_2_, C_2_H_4_, and CO_2_. One is in the interlaced channel and the other locates in the large cage. Moreover, all gas molecules prefer to be adsorbed in the interlaced channel with higher affinity. The realistic binding sites and host–guest interactions under normal conditions (298 K and 1.0 bar) were further demonstrated by in-situ single crystal structures with the saturated dose of gases. Notably, rare C_2_H_2_ clusters formed by π···π packing and C-H···C≡C interactions are observed in the interlaced channel with a small proportion of C_2_H_2_ molecules adsorbed in the large cage additionally. In sharp contrast, only 1/3 of CO_2_ molecules are located in the narrow channel while 2/3 of CO_2_ molecules are accommodated in the large cavity. This distinct gas distribution is highly consistent with the difference of adsorption heat curves. The practical C_2_H_4_ purification performance is further demonstrated by dynamic breakthroughs and record high C_2_H_4_ productivity is achieved from ternary C_2_H_2_/CO_2_/C_2_H_4_ mixtures in a single adsorption process under various conditions. The separation performance is retained over multiple cycles and under humid conditions.

## Results

**Fig. 1:**
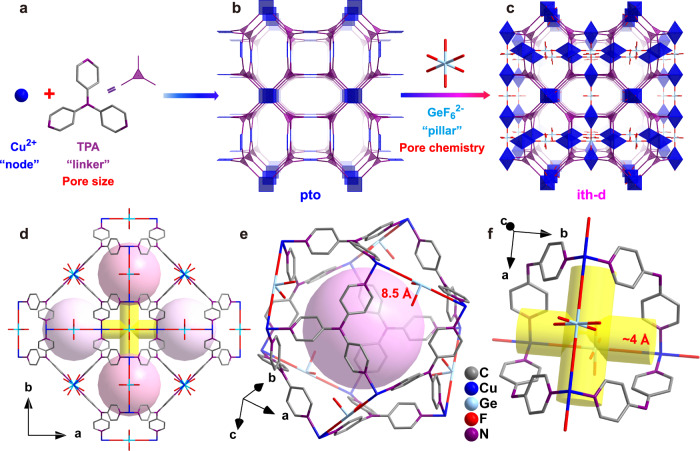
Porous structure of ZNU-6. **a–c** Exquisite control of pore size/shape and pore chemistry in ZNU-6 from pillared (3,4)-connected pto network to GeF_6_^2−^ embedded ith-d topology framework; **d** Overview of ZNU-6 structure with cage-like pores and interlaced channels. **e** Structure and size of the cage-like pore. **f** Structure and size of the interlaced channel connecting four cages.

Violet single crystals of ZNU-6 (Supplementary Fig. 1) were produced by layering a MeOH solution of TPA onto an aqueous solution of CuGeF_6_ (Fig. 1a)_._ X-ray crystal analysis revealed that ZNU-6 [Cu_6_(GeF_6_)_6_(TPA)_8_] crystallizes in a three-dimensional (3D) framework in the cubic Pm-3n space group. Every unit cell consists of six Cu^2+^ ions, six GeF_6_^2-^ anions, and eight tridentate TPA ligands (Supplementary Table 1). The combination of Cu^2+^ and TPA produces a cationic pto network first (Fig. 1b), which determines the main pore size. The network is further embedded by GeF_6_^2−^ pillar to give a ith-d topology framework with optimal pore chemistry (Fig. 1c). The frameworks are composed of large icosahedral cage-like pores (~8.5 Å) and interlaced narrow channels (~4 Å) (Fig. 1d–f). Each large cage is surrounded by 12 channels and every interlaced channel connects 4 cages. The adjacent two cages and two channels share the same GeF_6_^2-^ anions at the edge. Both large pores and interlaced channels are abundant of Lewis basic F functional sites on the surface for gas binding. Such interconnected large cages and narrow channels are distinct from previous straight 1D channels of anion pillared MOFs (e.g., SIFSIX-1-Cu, SIFSIX-3-Ni). Besides, the narrow channel size may provide kinetic selectivity for C_2_H_2_ (3.3 Å) and CO_2_ (3.3 Å) given their small molecular size compared to C_2_H_4_ (4.2 Å). Thus, ZNU-6 with abundant functional GeF_6_^2−^ binding sites, high porosity for C_2_H_2_ and CO_2_ accommodation and narrow channel for kinetic preference features the promising characteristics for efficient purification of C_2_H_4_ from ternary C_2_H_2_/CO_2_/C_2_H_4_ mixture.

**Fig. 2:**
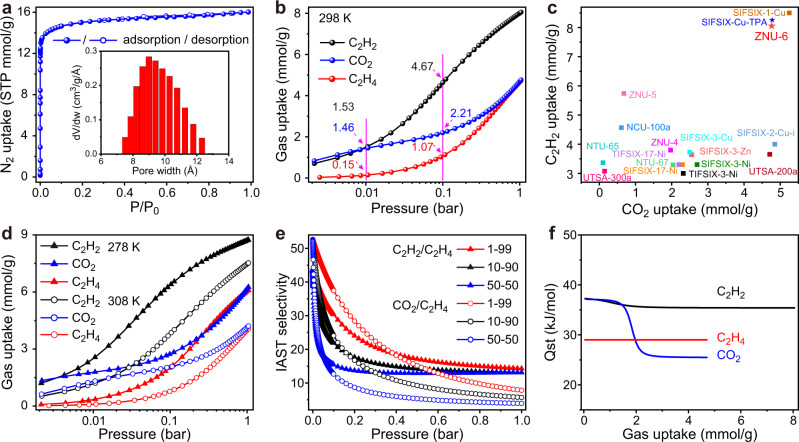
The sorption performance. **a** N_2_ adsorption and desorption isotherms for ZNU-6 and the calculated pore size distribution. **b** C_2_H_2_, CO_2_, and C_2_H_4_ adsorption isotherms of ZNU-6 at 298 K. **c** Comparison of the saturated C_2_H_2_ and CO_2_ uptake (1 bar, 298 K) among anion pillared MOFs. **d** C_2_H_2_, CO_2_, and C_2_H_4_ adsorption isotherms of ZNU-6 at 278/308 K. **e** C_2_H_2_/C_2_H_4_ and CO_2_/C_2_H_4_ IAST selectivity of ZNU-6 at 298 K. **f** *Q*_st_ of C_2_H_2_, CO_2,_ and C_2_H_4_ in ZNU-6. Source data are provided as a Source Data file.

The intrinsic porosity of ZNU-6 was investigated by N_2_ adsorption at 77 K. As shown in Fig. 2a, ZNU-6 exhibited a type I adsorption isotherm. The Brunauer–Emmett–Teller surface area and pore volume were calculated to be 1330.3 m^2^/g and 0.554 cm^3^/g (Supplementary Fig. 10). The calculated pore size ranges from 8.22 to 10.76 Å with the summit in 9.0 Å, highly close to the pore aperture of ~8.5 Å evaluated from the single crystal structure (Fig. 2a). Then, single-component adsorption isotherms of C_2_H_2_, CO_2_, and C_2_H_4_ were collected at 298 K (Fig. 2b). At 1.0 bar, the C_2_H_2_ and CO_2_ uptakes are 8.06 and 4.76 mmol/g, higher than those of most APMOFs (Fig. 2c). The capacities are equal to 4.68 and 2.77 gas molecules per GeF_6_^2−^ anion. Such high C_2_H_2_/anion and CO_2_/anion uptakes have never been realized in anion pillared MOFs (Supplementary Table S7)^36–38,40–44^. Particularly, C_2_H_2_/anion and CO_2_/anion uptakes in benchmark TIFSIX-17-Ni^36^, SIFSIX-17-Ni^36^ and NTU-67^38^ are only 1.36/0.91, 1.29/0.9 and 2.06/1.28, respectively (Supplementary Fig. 25). So far, isomorphic SIFSIX-Cu-TPA^40^ displays the ever highest C_2_H_2_/anion (4.44) uptake while SIFSIX-1-Cu^41^ displays the ever highest CO_2_/anion (2.72) uptake. It is worth mentioning that these records have been marginally surpassed by ZNU-6’s (Supplementary Fig. 25). Notably, the uptakes of C_2_H_2_ and CO_2_ on ZNU-6 at 0.01 bar are as high as 1.53 and 1.46 mmol/g, superior to those of all the porous materials in the context of ternary C_2_H_2_/CO_2_/C_2_H_4_ separation, such as TIFSIX-17-Ni (1.38/0.32 mmol/g)^36^, SIFSIX-17-Ni (0.91/0.20 mmol/g)^36^, NTU-67 (0.47/0.65 mmol/g)^38^, and TpPa-NO_2_ (0.17/0.03 mmol/g)^39^. At 0.1 bar, the capacities of C_2_H_2_ and CO_2_ reach up to 4.64 and 2.21 mmol/g (Fig. 2b), even higher than the uptakes of many porous materials at 1 bar and 298 K, for example, TIFSIX-17-Ni (3.30/2.20 mmol/g)^36^. In the meantime, the C_2_H_4_ uptakes on ZNU-6 at 0.01 and 0.1 bar are only 0.15 and 1.07 mmol/g, much lower than those of C_2_H_2_ and CO_2_ under the same conditions. The C_2_H_2_, CO_2_, and C_2_H_4_ adsorption isotherms were further collected at 278 and 308 K (Fig. 2d). The adsorption capacities of C_2_H_2_ and CO_2_ at 1 bar increase to 8.74 and 6.26 mmol/g at 278 K. As selectivity is also an important parameter to assess the separation performance, we further calculated the C_2_H_2_/C_2_H_4_ and CO_2_/C_2_H_4_ selectivities on ZNU-6 using ideal adsorbed solution theory (IAST) after fitting isotherms into dual site Langmuir or single site Langmuir equation with excellent accuracy. The IAST selectivity for 1/99 C_2_H_2_/C_2_H_4_ is 43.8–14.3 (Fig. 2e), higher than those of NTU-67 (8.1)^38^ and TpPa-NO_2_ (5.9)^39^. The IAST selectivities for 1/99 CO_2_/C_2_H_4_ mixture is also as high as 52.6-7.8 (Fig. 2e). Besides, both C_2_H_2_/C_2_H_4_ and CO_2_/C_2_H_4_ selectivity on ZNU-6 is improved with the pressure decrease or the increase of C_2_H_4_ ratios (from 90% to 99%) in the binary mixtures (Supplementary Figs. 13, 14), indicating ZNU-6 is favored for trace C_2_H_2_ and CO_2_ capture from bulky C_2_H_4_ mixtures. Apart from the IAST selectivity, the Henry coefficients were also calculated to evaluate the Henry’s selectivity of ZNU-6 (Supplementary Figs. 15–17), the Henry’s selectivity for C_2_H_2_/C_2_H_4_ and CO_2_/C_2_H_4_ is 8.2 and 7.8, respectively, superior to those of NTU-67 (2.4/4.2)^38^ and TpPa-NO_2_ (4.0/1.8)^39^ (Supplementary Tables 4, 5). We further calculated the isosteric enthalpy of adsorption (*Q*_st_) for ZNU-6 by using the Clausius-Clapeyron equation. *Q*_st_ values at near-zero loading for C_2_H_2_, CO_2_, and C_2_H_4_ are 37.2, 37.1, and 29.0 kJ/mol (Fig. 2f), respectively, indicative of the preferred affinity of C_2_H_2_ and CO_2_ over C_2_H_4_. Notably, the *Q*_st_ values for C_2_H_2_ and CO_2_ on ZNU-6 are only modestly high and slightly lower than those of many top-performing materials in the context of C_2_H_4_ purification, such as SIFSIX-17-Ni (44.2/40.2 kJ/mol)^36^, TIFSIX-17-Ni (48.3/37.8 kJ/mol)^36^, and NTU-67 (44.1/41.5 kJ/mol)^38^. Such moderate *Q*_st_ endows facile regeneration of ZNU-6 under mild conditions.

**Fig. 3:**
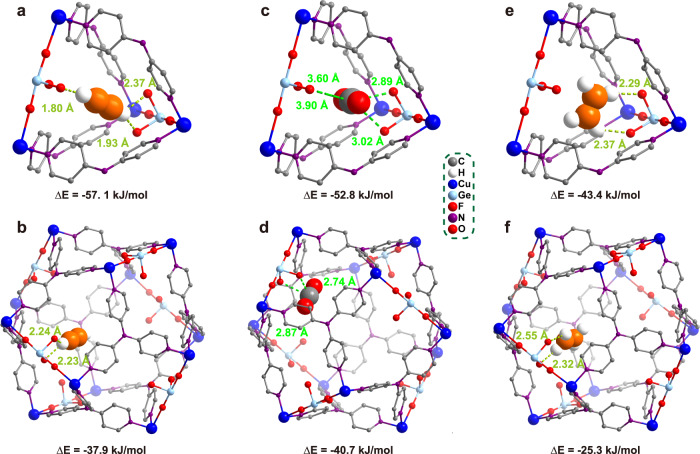
The DFT optimized gas adsorption configuration. Binding sites I, II of C_2_H_2_ (**a, b**), CO_2_ (**c, d**), and C_2_H_4_ (**e, f**).

To gain more insights into the gas adsorption behavior, density functional theory (DFT)-based calculations (see Method section) were applied to identify the adsorption configuration and binding energies of C_2_H_2_, CO_2_, and C_2_H_4_. For all gases, two different binding sites were observed. Site I is in the interlaced channel and Site II is in the large cavity (Fig. 3). For C_2_H_2_ in Site I, the two hydrogen atoms interact strongly with three F atoms with the distances of 1.80, 1.93, and 2.37 Å. The calculated binding energy is 57.1 kJ/mol (Fig. 3a). As for C_2_H_2_ adsorbed in Site II, only one hydrogen atom can interact with the adjacent F atoms with the distance of 2.23 and 2.24 Å, and the corresponding binding energy decreases to 37.9 kJ/mol (Fig. 3b), indicating that C_2_H_2_ is preferentially adsorbed in the narrow channel. The same results are also observed for CO_2_ and C_2_H_4_, the binding energies in the channel are much higher than those in the large cage. In Site I, CO_2_ is trapped by two strong and two weak electrostatic F···C=O interactions in the distance of 2.89, 3.02, 3.60, and 3.90 Å, the binding energy is 52.8 kJ/mol (Fig. 3c); C_2_H_4_ is adsorbed via two F···H interactions (2.29 and 2.37 Å) with the binding energy of 43.3 kJ/mol (Fig. 3e). In Site II, the binding energy of CO_2_ drops to 40.7 kJ/mol with the number of electrostatic F···C=O interactions (2.74 and 2.87 Å) decreasing to two (Fig. 3d); the binding energy of C_2_H_4_ reduces to 25.3 kJ/mol with the length of F···H extending to 2.55 and 2.32 Å (Fig. 3f). In addition, it is notable that either in Site I or II, the binding energy of C_2_H_2_ or CO_2_ is superior to that of C_2_H_4_, confirming that the adsorption of C_2_H_2_ or CO_2_ in ZNU-6 is more preferable than that of C_2_H_4_.

**Fig. 4:**
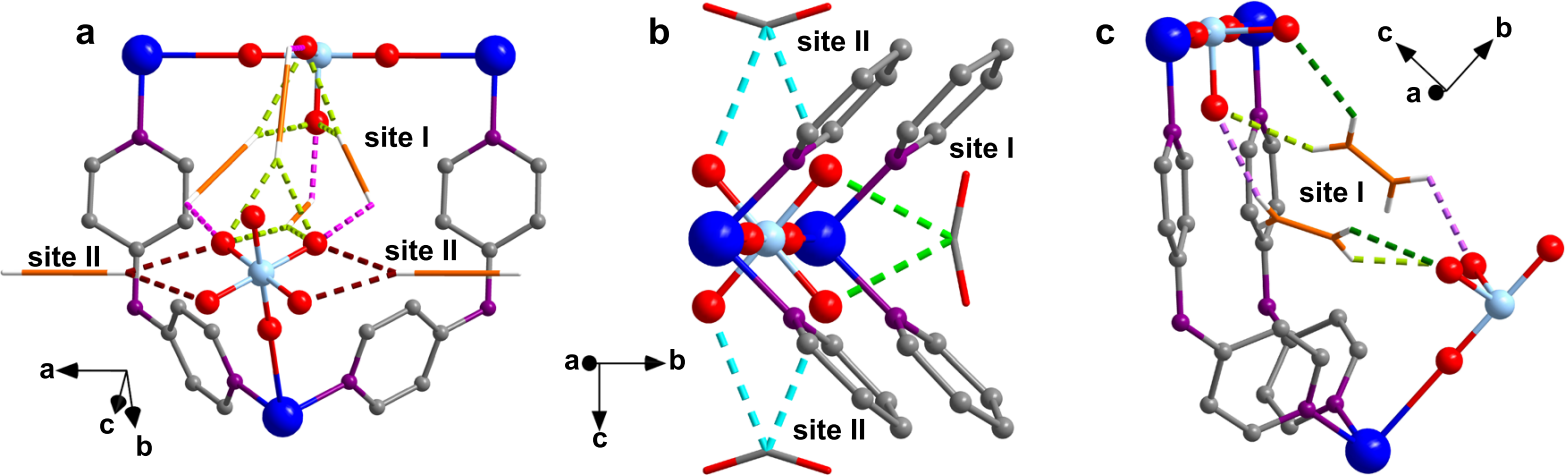
Single crystal structure of gas-loaded ZNU-6. **a** C_2_H_2_ @ ZNU-6 [Cu_6_(GeF_6_)_6_(TPA)_8_ (C_2_H_2_)_25.78_]; **b** CO_2_ @ ZNU-6 [Cu_6_(GeF_6_)_6_(TPA)_8_ (CO_2_)_18_]; **c** C_2_H_4_ @ ZNU-6 [Cu_6_(GeF_6_)_6_(TPA)_8_ (C_2_H_4_)_13.07_].

Although DFT calculations have identified two different binding sites for each gas, it is still difficult to understand the distinct adsorption heat curves. Therefore, we further studied the in-situ structures of ZNU-6 with gas loading (Fig. 4). We found that averagely 25.78 C_2_H_2_, 18 CO_2_, or 13.07 C_2_H_4_ molecules can be adsorbed per unit cell of ZNU-6 (Supplementary Table 1), corresponding to 4.3 C_2_H_2_, 3.0 CO_2_, and 2.2 C_2_H_4_ molecules for each GeF_6_^2-^ anion, which are close to the saturated values from gas adsorption isotherms (4.63 C_2_H_2_, 2.77 CO_2_, and 2.75 C_2_H_4_). Both of C_2_H_2_ and CO_2_ have two binding sites, i.e., Site I in the interlaced channel and Site II in the large cage. Notably, the amount of C_2_H_2_ molecules distributed to the two locations is 3.8 and 0.5 per GeF_6_^2-^ anion while that for CO_2_ is 1 and 2 per GeF_6_^2−^ anion (Fig. 4a, b). Such different gas distribution can precisely account for the C_2_H_2_ *Q*_st_ curve with a modest decrease and the CO_2_ *Q*_st_ curve with a sharp decrease along the gas loading. Specifically, C_2_H_2_ molecules adsorbed in Site I bind to F atoms on the surface of the channels via multiple cooperative hydrogen bonds (C-H···F = 1.97–2.55 Å), and the others in Site II interact F atoms via single H···F hydrogen bond with the distance of 2.51 Å (Fig. 4a and Table 1). Besides, the C_2_H_2_ molecules in Site I aggregate to form a stacked gas cluster by π···π packing and C-H···C≡C interactions, which has rarely been observed previously. Regarding CO_2_, it is trapped by F···C=O electrostatic interaction in Site I and II (Fig. 4b). The only difference is that the C···F distance is 2.64 Å in Site I and 2.80 Å in Site II (Table 1). From the single crystal structure, two different CO_2_ molecules that are very close and opposite to each other in the narrow channel (site I) are observed. However, these two CO_2_ molecules cannot exist in the same narrow channel at the same time and thus both CO_2_ molecules display the occupancy of 50%. In Site II, the C atom of CO_2_ is ordered while the O atoms are disordered to two perpendicular positions with the occupancy of 50% for each configuration. Besides, the linear CO_2_ molecules are slightly bent due to the strong attraction from GeF_6_^2−^ anion. The bent angle of 157.5° (in Site I) and 170.8° (in Site II) are consistent with the interaction strength. In term of C_2_H_4_, only one site in the narrow channel is found. The C atoms of C_2_H_4_ molecule are ordered while the H atoms are disordered. The distances of C-H···F interactions between C_2_H_4_ and framework are 2.31–2.64 Å (Fig. 4c and Table 1). Considering the slight lower C_2_H_4_/GeF_6_^2−^ ratio observed in the single crystal structure, there should be some C_2_H_4_ molecules adsorbed in the large cage. However, due to the probable disorder of C_2_H_4_ molecules over the whole cage, the C_2_H_4_ molecules in Site II were not solved. Nonetheless, this uniform adsorption configuration is consistent with the flat *Q*_st_ curve for C_2_H_4_.

**Table 1:** The distances of the host–guest interactions

Crystals	Site I	Site II
25.78 C_2_H_2_ @ ZNU-6	1.97/2.55 Å (C-H···F)	2.51 Å (C-H···F)
18 CO_2_ @ ZNU-6	2.64 Å (C···F)	2.80 Å (C···F)
13.07 C_2_H_4_ @ ZNU-6	2.31/2.54/2.64 Å (C-H···F)	-

Apart from the C_2_H_2_, CO_2_, or C_2_H_4_ molecules, some water molecules were also identified in the framework (Supplementary Fig. 4). As there is still a lot of space in the large cavity after saturated adsorption of C_2_H_2_, CO_2_, or C_2_H_4_ gases at 100 kPa, the water adsorption behavior probably occurred during the single crystal measurement, which is exposed to air. Interestingly, these water molecules are distant from GeF_6_^2-^_,_ indicating that these H_2_O molecules do not occupy the binding sites for the targeted gases. Instead, some unique interactions are observed between the gas molecules and water molecules, e.g., O-H···C=O hydrogen bonds between CO_2_ and H_2_O. Notably, our resolved single crystal structures show completely different C_2_H_2_ and CO_2_ adsorption configurations from those of isomorphic SIFSIX-Cu-TPA for C_2_H_2_/CO_2_ separation in Wu’s work^39^.

**Fig. 5:**
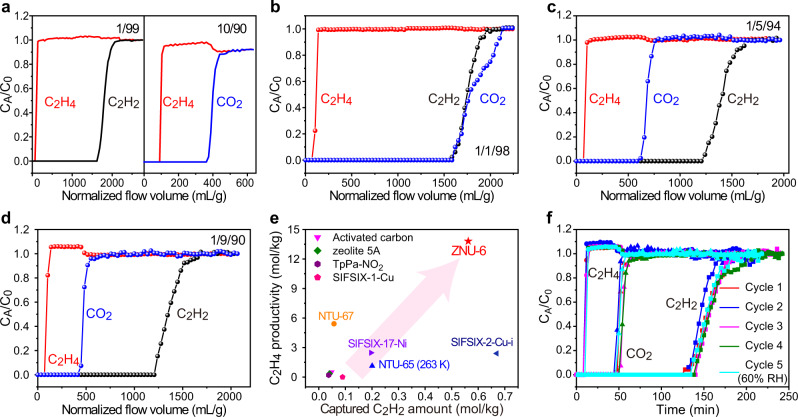
C_2_H_4_ purification. Experimental breakthrough curves of ZNU-6 for binary mixture **a** C_2_H_2_/C_2_H_4_ (1/99) and CO_2_/C_2_H_4_ (10/90) at 298 K. Experimental breakthrough curves of ZNU-6 for ternary mixture **b** C_2_H_2_/CO_2_/C_2_H_4_ (1/1/98), **c** C_2_H_2_/CO_2_/C_2_H_4_ (1/5/94), and **d** C_2_H_2_/CO_2_/C_2_H_4_ (1/9/90). **e** Comparison of the captured C_2_H_2_ amount and C_2_H_4_ productivity from C_2_H_2_/CO_2_/C_2_H_4_ (1/9/90) ternary mixture. **f** Five cycles of experimental breakthrough curves of ZNU-6 for C_2_H_2_/CO_2_/C_2_H_4_ (1/9/90) at 298 K (1–4: dry condition, 5: humid condition). Source data are provided as a Source Data file.

Motivated by the high adsorption capacity and selectivity in single-component adsorption as well as the in-situ single crystal structure analysis, breakthrough experiments were conducted for C_2_H_2_/C_2_H_4_, CO_2_/C_2_H_4_, and C_2_H_2_/CO_2_/C_2_H_4_ mixtures. The results showed that highly efficient separations can be accomplished by ZNU-6 for all the gas mixtures under various conditions. For 1/99 C_2_H_2_/C_2_H_4_ mixtures, C_2_H_4_ is eluted at 12 mins while C_2_H_2_ is detected until 192 min. For 10/90 CO_2_/C_2_H_4_ mixtures, C_2_H_4_ and CO_2_ are detected at 12 and 43.5 min, respectively (Fig. 5a). For 1/1/98 C_2_H_2_/CO_2_/C_2_H_4_ mixtures, C_2_H_2_ and CO_2_ broke out simultaneously and 64.42 mol/kg of polymer grade C_2_H_4_ is produced by single adsorption process (Fig. 5b). The productivity is improved to 80.89 mol/kg when decreasing the temperature to 283 K (Supplementary Fig. 46). The CO_2_ breakthrough time becomes shortened with the increase of CO_2_ ratio, which is 72 and 52 min for 1/5/94 (Figs. 5c) and 1/9/90 (Fig. 5d) C_2_H_2_/CO_2_/C_2_H_4_ mixtures. The polymer grade C_2_H_4_ productivity is 21.37 and 13.81 mol/kg, respectively. As most reported C_2_H_4_ productivity from C_2_H_2_/CO_2_/C_2_H_4_ mixtures are compared under 1/9/90, a comparison plot of the C_2_H_4_ productivity and dynamic C_2_H_2_ capacity from 1/9/90 C_2_H_2_/CO_2_/C_2_H_4_ mixtures is presented in Fig. 5e. ZNU-6 displays the record high C_2_H_4_ productivity and second highest C_2_H_2_ dynamic capacity. The C_2_H_4_ productivity of ZNU-6 is >2.5 folds of the previous benchmark of NTU-67 (5.42 mol/kg)^38^. C_2_H_4_ productivity with the unit of mol/kg/h is also calculated for comparison (Supplementary Table S10). ZNU-6 with the productivity of 15.93 mol/kg/h is the highest reported value.

In view of the importance of the recyclability and stability of porous materials for practical applications, the water and thermal stability of ZNU-6 was investigated. There was no noticeable loss in the CO_2_ adsorption capacity after six cycles of adsorption/desorption experiments (Supplementary Fig. 26). Long time soaking of ZNU-6 in water or polar organic solvents such as DMSO, DMF and MeCN did not change the porous structure of ZNU-6, as demonstrated by the PXRD patterns as well as the gas adsorption isotherms (Supplementary Fig. 7). Thermogravimetric analysis (TGA) and temperature varied PXRD indicated ZNU-6 is stable below 200 °C (Supplementary Figs. 8, 9). Breakthroughs under humid conditions or over four cycles preserved nearly the identical separation performance (Fig. 5f). Although many water molecules can be adsorbed in ZNU-6, as described in in-situ crystals and water adsorption isotherms (Supplementary Fig. 27), the presence of humid has negligible influence on the separation performance (Fig. 5f). This is probably due to the co-adsorption of water and target gases as well as the fast C_2_H_2_/CO_2_/C_2_H_4_ diffusion kinetics (Supplementary Fig. 29–31).

## Discussion

In conclusion, we reported a GeF_6_^2−^ anion embedded metal organic framework ZNU-6 with optimal pore structure and pore chemistry for benchmark one-step C_2_H_4_ purification by simultaneous removal of C_2_H_2_ and CO_2_. ZNU-6 exhibits remarkably high C_2_H_2_ and CO_2_ capacity under both low and high pressures. The C_2_H_2_/anion and CO_2_/anion uptakes are the highest among all the anion pillared MOFs. 64.42, 21.37, 13.81 mol/kg polymer grade C_2_H_4_ can be produced from C_2_H_2_/CO_2_/C_2_H_4_ (1/1/98, 1/5/94, 1/9/90) mixtures, all superior to the previous benchmarks. The separation performance is sustained over multiple cycles or under humid conditions. The potential gas binding sites are investigated by DFT calculation, which indicate that C_2_H_2_ and CO_2_ are preferentially adsorbed in the interlaced narrow channel with high affinity. In-situ single crystal structures with the dose of C_2_H_2_, CO_2_ or C_2_H_4_ further reveal the realistic host–guest interactions, accounting for the distinct shapes of the adsorption heat curves. In general, our work highlights the significance of regulating pore structure and pore chemistry in porous materials to construct multiple cooperative functionalities for gas separation.

## Methods

### Synthesis of ZNU-6

To a 5 mL long thin tube was added a 1 mL of aqueous solution with Cu(NO_3_)_2_·3H_2_O (~1.3 mg) and (NH_4_)_2_GeF_6_ (~1.0 mg). 2 mL of MeOH/H_2_O mixture (v:v = 1:1) was slowly layered above the solution, followed by a 1 mL of MeOH solution of TPA (~1.0 mg). The tube was sealed and left undisturbed at 298 K. After ~1 week, purple single crystals were obtained.

### Preparation of gas loaded ZNU-6

The crystalline sample of ZNU-6 was filled into a glass tube and heated at 120 °C under vacuum for 24 h. After the sample cooling down, CO_2_, C_2_H_2_, or C_2_H_4_ was introduced into the sample respectively with Builder SSA 7000 (Beijing) instrument until the pressure reach to 1 bar at 298 K and the state is maintained for another hour. Then, the crystals were picked out, covered with the degassed oil, and single crystal X-ray diffraction measurements were then carried out at 298 K as soon as possible.

### Single-crystal X-ray diffraction

Single-crystal X-ray diffraction studies were conducted on the BrukerAXS D8 VENTURE diffractometer equipped with a PHOTON-100/CMOS detector (GaKα, *λ* = 1.34139 Å). Indexing was performed using APEX2. Data integration and reduction were completed using SaintPlus 6.01. Absorption correction was performed by the multi-scan method implemented in SADABS. The space group was determined using XPREP implemented in APEX2.1 The structure was solved with SHELXS-97 (direct methods) and refined on F2 (nonlinear least-squares method) with SHELXL-97 contained in APEX2, WinGX v1.70.01, and OLEX2 v1.1.5 program packages. All non-hydrogen atoms were refined anisotropically. The contribution of disordered solvent molecules was treated as diffuse using the Squeeze routine implemented in Platon.

### Powder X-ray diffraction

Powder X-ray diffraction (PXRD) data were collected on the SHIMADZU XRD-6000 diffractometer (Cu Kα*λ* = 1.540598 Ǻ) with an operating power of 40 kV, 30 mA and a scan speed of 4.0°/min. The range of 2*θ* was from 5° to 50°.

### Thermal gravimetric analysis

Thermal gravimetric analysis was performed on the TGA STA449F5 instrument. Experiments were carried out using a platinum pan under nitrogen atmosphere which conducted by a flow rate of 60 mL/min nitrogen gas. The data were collected at the temperature range of 50 °C to 600 °C with a ramp of 10 °C /min.

### The static gas/vapor adsorption equilibrium measurements

The static gas adsorption equilibrium measurements were performed on the Builder SSA 7000 instrument. The water vapor adsorption equilibrium measurements were performed on the BeiShiDe DVS instrument. Before measurements, the sample of ZNU-6 (~100 mg) was evacuated at 25 °C for 2 h firstly, and then at 120 °C for 12 h until the pressure dropped below 7 μmHg. The sorption isotherms were collected at 77 K, 278, 298, and 308 K on activated samples. The experimental temperatures were controlled by liquid nitrogen bath (77 K) and water bath (278, 298, and 308 K), respectively.

### Breakthrough experiments

The breakthrough experiments were carried out on a dynamic gas breakthrough equipment. The experiments were conducted using a stainless steel column (4.6 mm inner diameter × 50 mm length). The weight of ZNU-6 powder packed in the columns were 0.5806 g. The column was activated at 75 °C for 2 h under vacuum, and then raised to 120 °C for overnight. The mixed gas of C_2_H_2_/C_2_H_4_ (1/99, v/v), CO_2_/C_2_H_4_ (10/90, v/v), or C_2_H_2_/CO_2_/C_2_H_4_ (1/9/90, 1/5/94, 1/1/98, 5/5/90, v/v/v) was then introduced. C_2_H_2_/CO_2_/C_2_H_4_ mixtures are produced by mixing three pure gases or mixing binary mixture with pure gas. Every flowrate was calibrated by self-made soap film flowmeter. Outlet gas from the column was monitored using gas chromatography (GC-9860-5CNJ) with the thermal conductivity detector TCD. After the breakthrough experiment, the sample was regenerated with an Ar flow of 5 mL min^−1^ under 120 °C for 8 h or under vacuum at 120 °C for 8 h.

### Fitting of experimental data on pure component isotherms

The unary isotherms for C_2_H_2_ and CO_2_ measured at three different temperatures 278 K, 298 K, and 308 K in ZNU-6 were fitted with excellent accuracy using the dual-site Langmuir model, where we distinguish two distinct adsorption sites A and B:

1 $$q=\frac{{q}_{{sat},A}{b}_{A}p}{1+{b}_{A}p}+\frac{{q}_{{sat},B}{b}_{B}p}{1+{b}_{B}p}$$

In Eq (S1), the Langmuir parameters ${b}_{A},{b}_{B}$ are both temperature dependent

2 $${b}_{A}={b}_{A0}\exp \left(\frac{{E}_{A}}{{RT}}\right);{b}_{b}={b}_{B0}\exp \left(\frac{{E}_{B}}{{RT}}\right)$$

In Eq. (2), ${E}_{A},{E}_{B}$ are the energy parameters associated with sites A, and B, respectively.

The corresponding unary isotherms for C_2_H_4_ measured at three different temperatures 278 K, 298 K, and 308 K in ZNU-6 were fitted with excellent accuracy using the single-site Langmuir model.

3 $$q=\frac{{q}_{{sat},A}{b}_{A}p}{1+{bp}}$$

The unary isotherm fit parameters for C_2_H_2_, CO_2_, and C_2_H_4_ are provided in Table S1.

### IAST calculations

The adsorption selectivity for separation of binary mixtures of species 1 and 2 is defined by

4 $${S}_{{ads}}=\frac{{q}_{1}/{q}_{2}}{{p}_{1}/{p}_{2}}$$

where *q*_1_, *q*_2_ are the molar loading (units: mol kg^-1^) in the adsorbed phase in equilibrium with a gas mixture with partial pressures *p*_1_, *p*_2_ in the bulk gas.

### Calculation of isosteric heat of adsorption (*Q*_st_)

The isosteric heat of adsorption, *Q*_st_, is defined as

5 $${Q}_{{st}}=-R{T}^{2}{\left(\frac{\partial \,{{{{\mathrm{ln}}}}}\,p}{\partial T}\right)}_{q}$$

where the derivative in the right member of Eq. (5) is determined at constant adsorbate loading, *q*. The calculations are based on the Clausius-Clapeyron equation.

### Density functional theory calculation

In this work, the DFT-based calculations were carried out using the CP2K package^45^. The Perdew-Burke-Ernzerhof (PBE) exchange functional^46^, Gaussian plane wave (PAW) pseudopotentials^47^ and DZVP basis sets^48^ for carbon, oxygen, fluorine, nitrogen, germanium and copper atoms, were used to describe the exchange–correlation interactions and electron–ion interaction, respectively. At the same time, the PBE-D3 method^49^ with Becke–Jonson damping for all atoms and Hubbard U corrections for the open-shell 3d transition metal (Cu) was used for geometry optimizations. The *U* value of 5.0 eV was used in this study. In all calculations, the net charges of simulation systems were set to zero. The adsorption energy can be obtained from formula below:

6 $${E}_{{ads}}={E}_{{adsorbate}+{substrate}}-{E}_{{substrate}}-{E}_{{adsorbate}}$$

where *E*_adsorbate+substrate_ and *E*_substrate_ were the total energies of the substrate with and without adsorbate, and *E*_adsorbate_ was the energy of the adsorbate.

## Supplementary information

Supplementary Information

Peer Review File

## Data Availability

The authors declare that the data supporting the findings of this study are available within the article and Supplementary Information. The X-ray crystallographic data related to ZNU-6 have been deposited at the Cambridge Crystallographic Data Centre (CCDC), under deposition numbers 2192744–2192747, respectively. These data can be obtained free of charge from the CCDC via www.ccdc.cam.ac.uk/data_request/cif. The data that support the findings of this study are available from the corresponding author. Besides, Source data are provided with this paper.

## Source data

Source data
