# Supplementary material for: Benchmark single-step ethylene purification from ternary mixtures by a customized fluorinated anion-embedded MOF
Source: Nat Commun. 2023 Jan 25;14:401. doi: 10.1038/s41467-023-35984-5 (PMC9876924; doi:10.1038/s41467-023-35984-5)
Supplement: Supplementary file 1 — Supplementary Information [file 41467_2023_35984_MOESM1_ESM.pdf]

Supplementary information

Benchmark Single-Step Ethylene Purification from Ternary Mixtures by a Customized Fluorinated Anion Embedded MOF

Jiang et al

**I**	**General Information and Procedures**	**p. 3**
**II**	**Characterization (SCXRD, PXRD, TGA, IR)**	**p. 4–9**
**III**	**Adsorption data, Selectivity and Q_{st}**	**p. 10–28**
**IV**	**Kinetic studies**	**p. 29–32**
**V**	**Breakthrough experiments**	**p. 33–44**
**VI**	**References**	**p. 45–46**

I General Information and Procedures

Unless otherwise noted, all the reactions were performed under air without N_2 or Ar protection. All reagents were used as received without purification unless stated otherwise.

Chemicals: Tri(pyridin-4-yl)amine (TPA, 99%), $Cu(NO_3)_2 \cdot 3H_2O$ (99%) and $(NH_4)_2GeF_6$ (99.99%) were purchased from Energy Chemical. C_2H_2 (99.9%), C_2H_4 (99.9%), CO_2 (99.99%), N_2 (99.9999%), He (99.9999%), Ar (99.9999%), C_2H_2/C_2H_4 (1:99), CO_2/C_2H_4 (10:90), C_2H_2/CO_2 (50:50) were purchased from Datong Co., Ltd.

II Characterization (SCXRD, PXRD, TGA, IR)

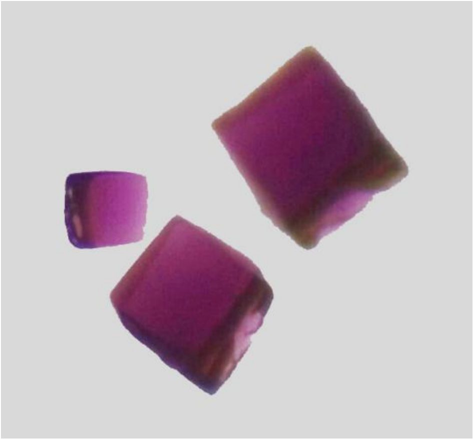

Supplementary Figure 1. Photography of the single crystals of **ZNU-6**.

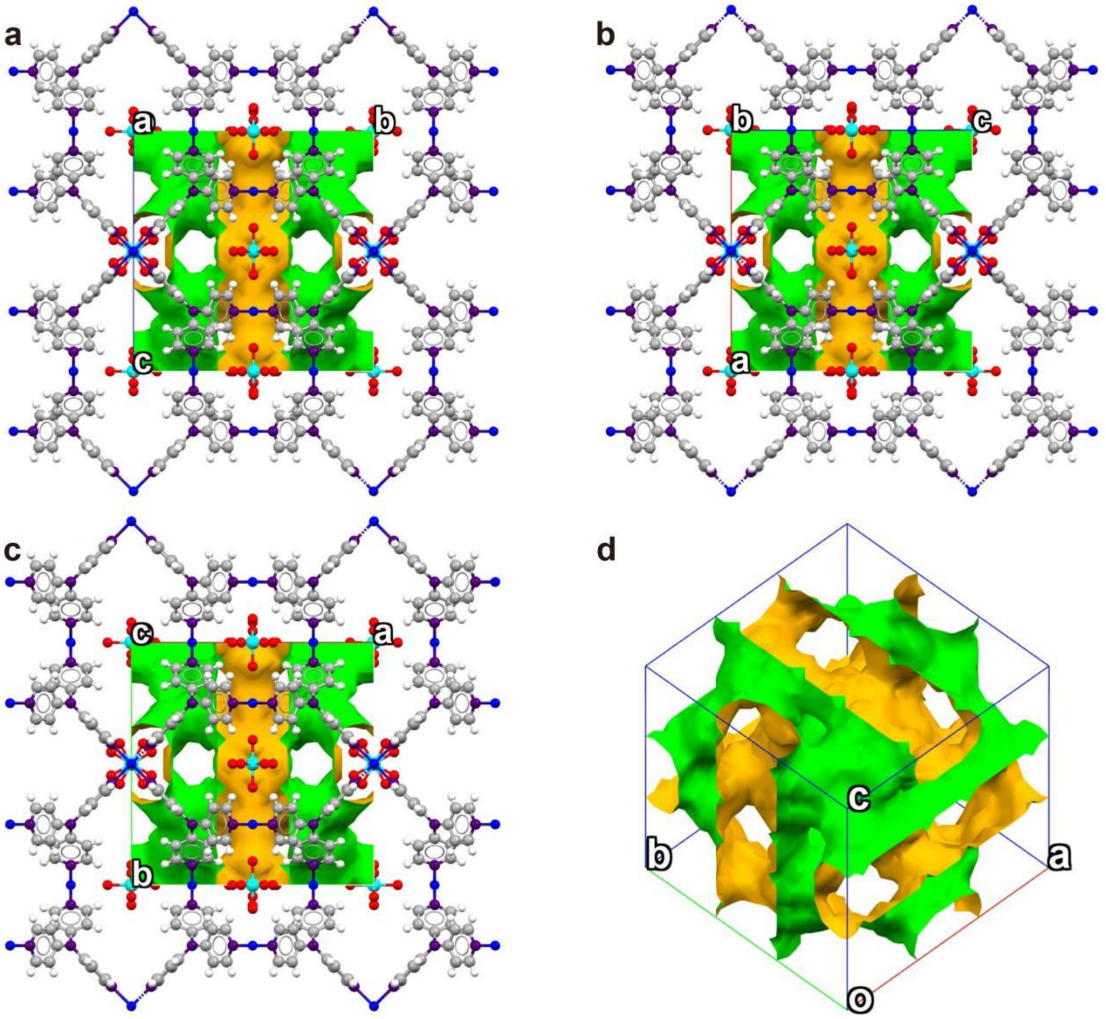

Supplementary Figure 2. 2 × 2 × 2 packing diagrams of **ZNU-6** viewed along the crystallographic *a*-, *b*-, and *c*-axis (a, b, c) and 1 × 1 × 1 packing diagrams of **ZNU-6** with pore surface in green representing the inside and yellow the outside determined using a probe with the radius of 1.2 Å by PLATON.

Supplementary Table 1. Single crystal data of as synthesized ZNU-6, activated ZNU-6, ZNU-6·C_2H_2, ZNU-6·C_2H_4 and ZNU-6·CO_2

		ZNU-6<br>as synthesized	ZNU-6·C_2H_2	ZNU-6·C_2H_4	ZNU-6·CO_2
cell	a=b=c	17.5352(3)	17.5343(3)	17.5392(3)	17.5395(2)
	α=β=γ	90°	90°	90°	90°
Temperature		298 K	298 K	298 K	298 K
Space group		Pm-3n	Pm-3n	Pm-3n	Pm-3n
Hall group		-P4n23	-P4n23	-P4n23	-P4n23
Formula		$C_{20}H_{16}Cu$<br>$F_6GeN_{5.33}$<br>$Cu(GeF_6)(TPA)_{1.33}$	$C_{20}H_{16}CuGeF_6$<br>$N_{5.33}\cdot 4.296C_2H_2$<br>$Cu(GeF_6)(TPA)_{1.33}$ $(C_2H_2)_{4.296}$	$C_{20}H_{16}CuGeF_6$<br>$N_{5.33}\cdot 2.178C_2H_4$<br>$Cu(GeF_6)(TPA)_{1.33}$<br>$(C_2H_4)_{2.178}$	$C_{20}H_{16}CuGeF_6$<br>$N_{5.33}\cdot 3CO_2$<br>$Cu(GeF_6)(TPA)_{1.33}$ $(CO_2)_3$
MW		581.21	701.29	660.38	767.85
density		1.074	1.296	1.219	1.418
Data completeness		0.988	0.985	0.967	0.971
R		0.1146	0.1460	0.1314	0.1451
wR2		0.2588	0.3304	0.2816	0.2970
S		0.986	1.201	1.059	1.002
CCDC. No		2192744	2192745	2192746	2192747

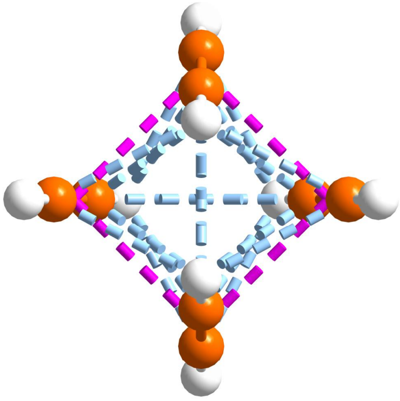

Supplementary Figure 3. The adsorption configuration of C_2H_2 moleucles inside the norrow channel (site I) of ZNU-6 with the formation of rare C_2H_2 clusters.The C-H interaction and π···π packing distance is highlighted.

There are two kinds of interactions between C_2H_2 molecules in the site I. One is the C···H interactions, whose distances are between 2.2 and 2.6 Å, and the other is π···π interactions between C≡C bonds, which are all in the distance of 2.4 Å.

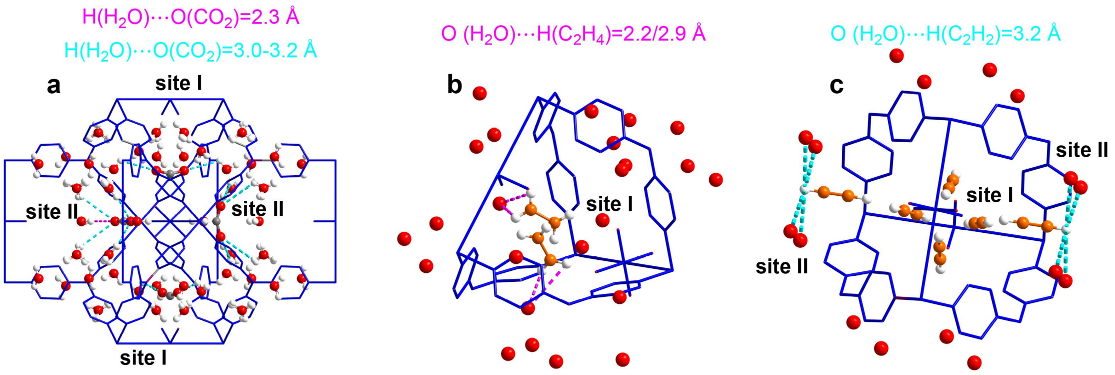

Supplementary Figure 4. Single crystals structure of gas loaded ZNU-6. **a**. $CuGeF_6C_{20}H_{16}N_{5.33}$ $(CO_2)_3$ $(H_2O)_{3.274}$. **b**. $CuGeF_6C_{20}H_{16}N_{5.33}$ $(C_2H_4)_{2.178}$ $(O)_{1.137}$. **c**. $CuGeF_6C_{20}H_{16}N_{5.33}$ $(C_2H_2)_{4.296}$ $(O)_{0.517}$.

Due to the serious disorder of H atoms of H_2O molecules, we haven't solve the H atoms in C_2H_2 and C_2H_4 loaded ZNU-6. In CO_2 loaded crystal, besides 18 CO_2 molecules, there are 19.644 water molecules in each unit cell (sum formula $Cu_6Ge_6F_{36}C_{120}H_{96}N_{32}$). As to C_2H_4 loaded crystals, there are 13.068 C_2H_4 molecules and 6.822 H_2O molecules in an unit cell. In the C_2H_2 loaded crystals, the number of H_2O (3.102) is much lower than that of CO_2 or C_2H_4 loaded crystals. These H_2O vapor molecules don't occupy the adsorption site of targeted gas molecules. Instead, some weak interactions between H_2O and targeted gas molecules were observed. As shown above, The distances of H (H_2O) and O (CO_2) are 2.3-3.2 Å, those of O (H_2O) and H (C_2H_4) are 2.2 and 2.9 Å, and those of O (H_2O) and H (C_2H_2) are 3.2 Å.

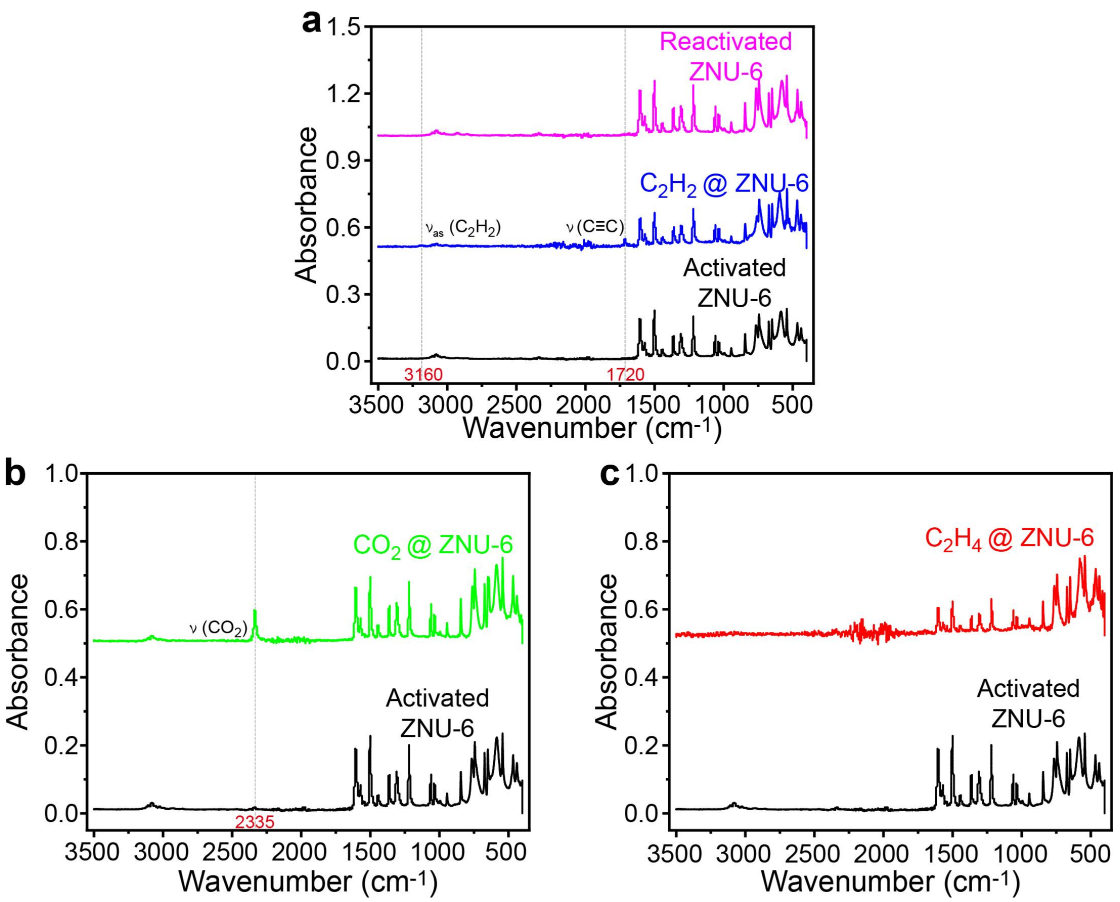

Supplementary Figure 5. In-situ IR spectra for **a.** activated ZNU-6 (black), -C_2H_2@ZNU-6 (blue) and re-activated ZNU-6 (purple); **b.** activated ZNU-6 (black) and CO_2@ZNU-6 (green); **c.** activated ZNU-6 (black) and C_2H_4@ZNU-6 (red).

All the IR spectroscopic data are recorded in a Nicolet iS5 ATR-FTIR spectrometer. The samples of gas-loaded crystals were prepared by the method described in **Preparation of gas loaded ZNU-6** in manuscript.

As shown in the Supplementary Figure 5, new and obvious stretching bands that belong to C_2H_2 and CO_2 are observed in the C_2H_2 and CO2 dosed single crystals. The $\nu_{as}(C_2H_2)$ and ν(C≡C) stretching band of adsorbed C_2H_2 down-shifted to 3160 and 1720 cm^{-1} respectively with reference to the gas-phase value at 3287 and 2500-1900 cm^{-1}, indicating the existence of guest-host interactions. Similarity, $\nu(CO_2)$ band also undergoes a downward shift from gas-phase value 2349 cm^{-1} to 2335 cm^{-1}, showing the interactions between CO_2 and framework. In contrast, the stretching band of C_2H_4 is not obvious in C_2H_4@ZNU-6.

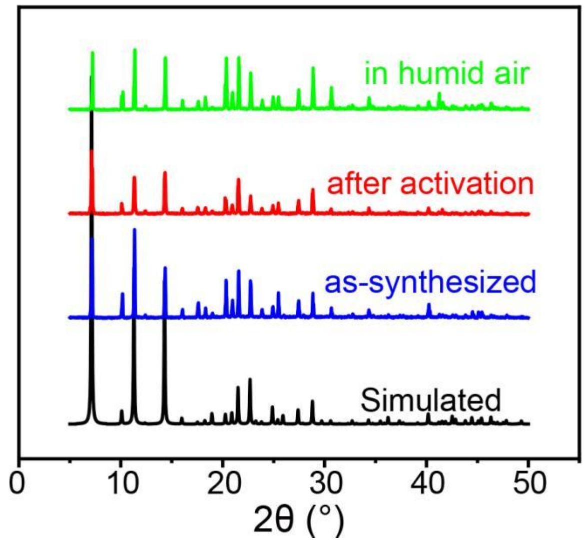

Supplementary Figure 6. PXRD patterns of **ZNU-6**.

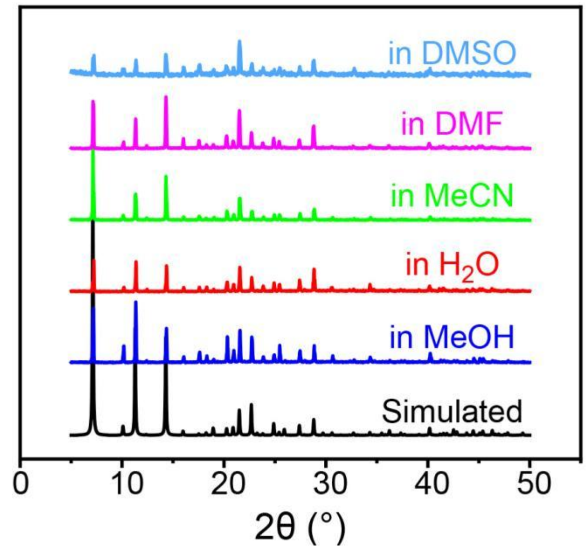

Supplementary Figure 7. PXRD patterns of **ZNU-6** after soaking in solvents for 6 months.

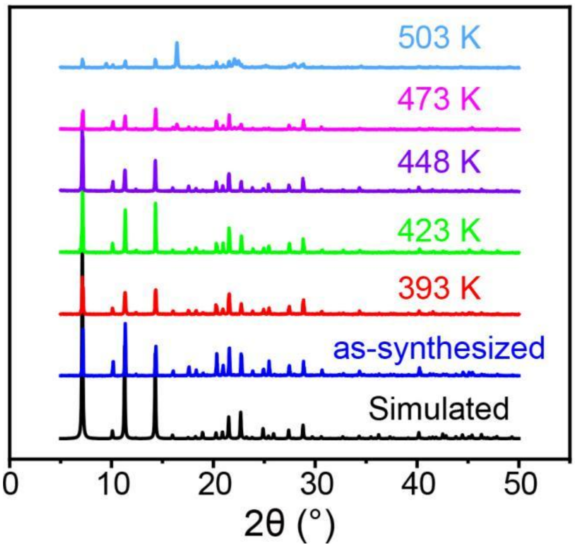

Supplementary Figure 8. PXRD patterns of **ZNU-6** after treatment under different temperatures.

Experimental method: The fresh samples of **ZNU-6** were evacuated at 25 ºC for 2 h firstly, and then evacuated at the corresponding temperature (393/423/448/473/503 K) for 40 mins. After cooling to room temperature, PXRD data were collected.

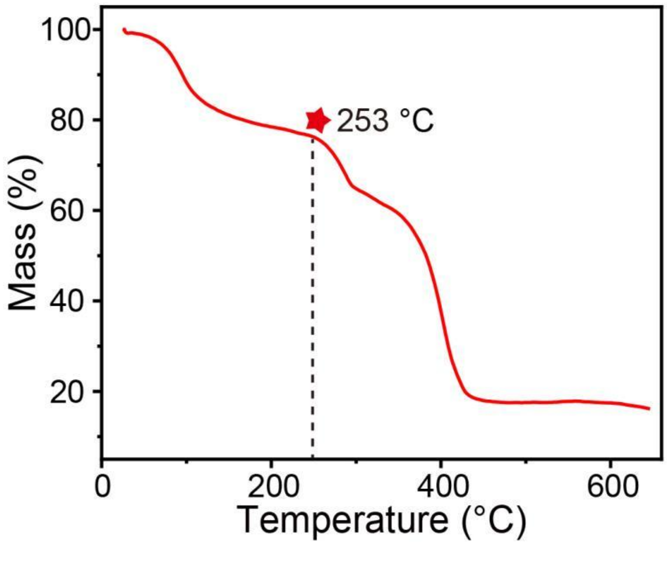

Supplementary Figure 9. TGA curve of **ZNU-6**. The weight loss before 110 ºC is because of the loss of MeOH and water from the sample. The weight keeps consistent until ~253 ºC.

III Adsorption data, Selectivity and Q_{st}

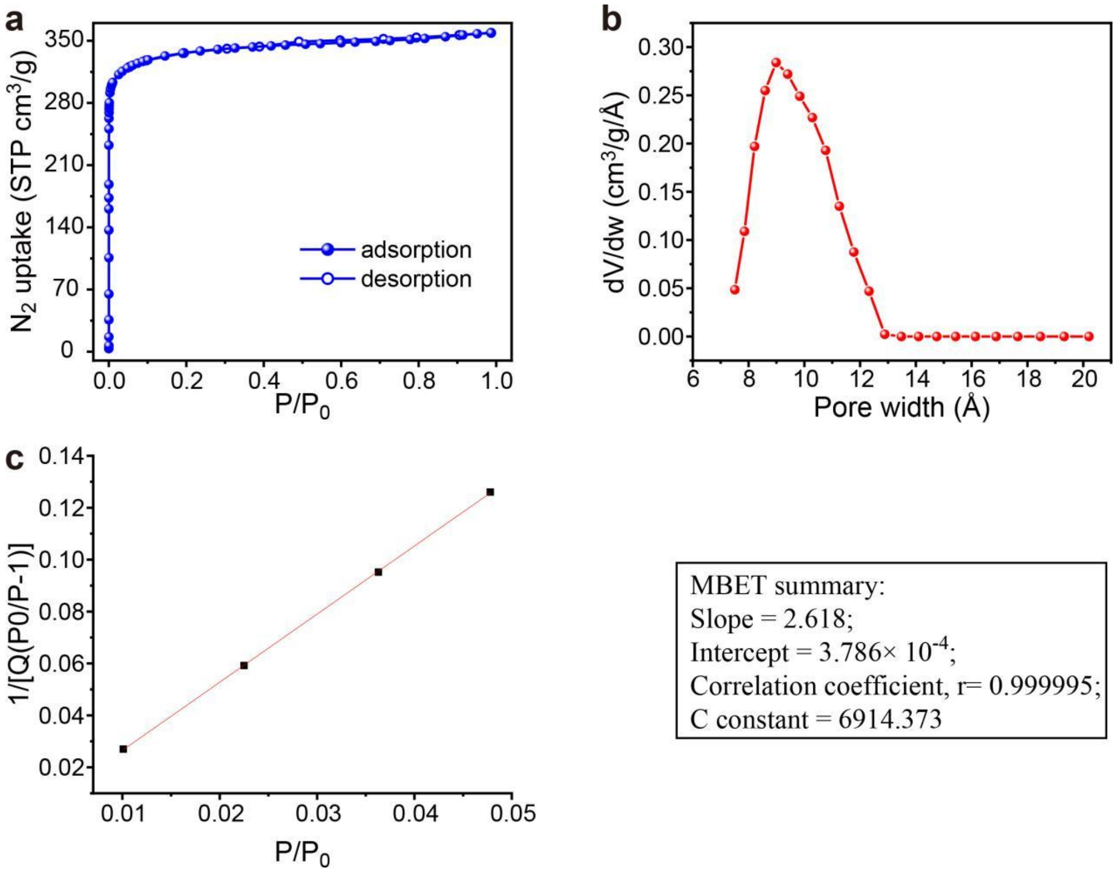

Supplementary Figure 10. Pore size distribution (b) of **ZNU-6** calculated from 77 K N_2 adsorption isotherms (a, c).

The BET surface area calculated from the N_2 adsorption isotherms under the pressure range of P/P_0 = 0.01-0.05 (for micropores) is 1330.3 m^2/g.

The total pore volume calculated from the N_2 adsorption isotherms is 0.554 cm^3/g.

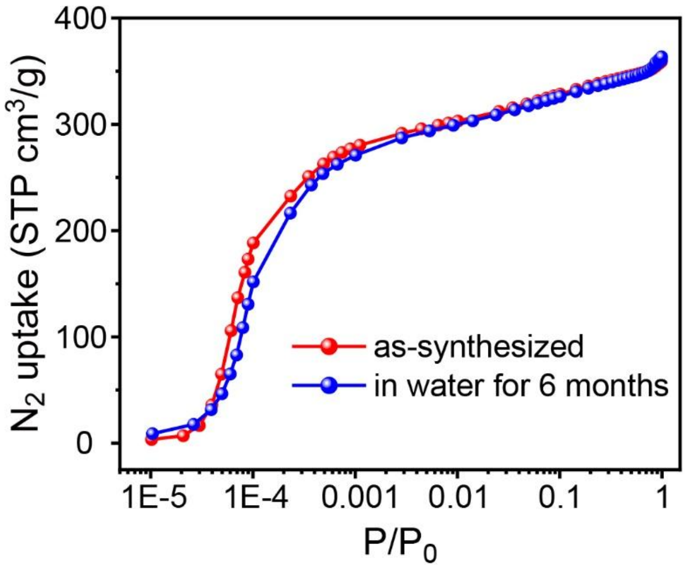

Supplementary Figure 11. The adsorption isotherm of N_2 at 77 K on as-synthesized **ZNU-6**, and **ZNU-6** after soaking in water for 6 months.

Analysis: The overlapping of the N_2 adsorption isotherms suggests its good stability towards water.

Supplementary Table 2. Comparison of C_2H_2, C_2H_4 and CO_2.

Gas molecules	Kinetic Diameter (Å)	Molecular size (Å^3)	Boiling point (K)	Polarizability ($\times 10^{-25}$ cm^3)
C_2H_2	3.3	3.32 x 3.34 x 5.70	189.3	33.3-39.3
C_2H_4	4.2	3.28 x 4.18 x 4.84	169.5	42.5
CO_2	3.3	3.18 x 3.33 x 5.36	194.7	25.93

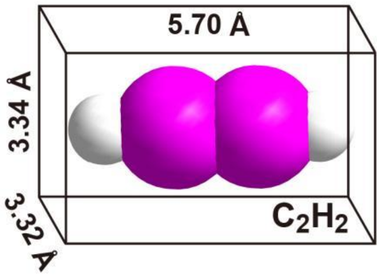

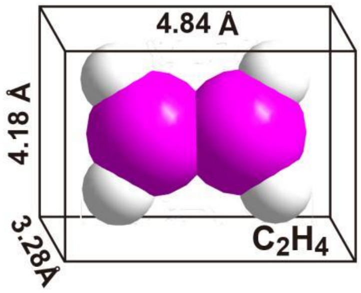

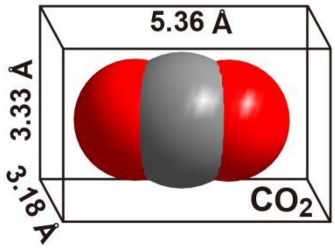

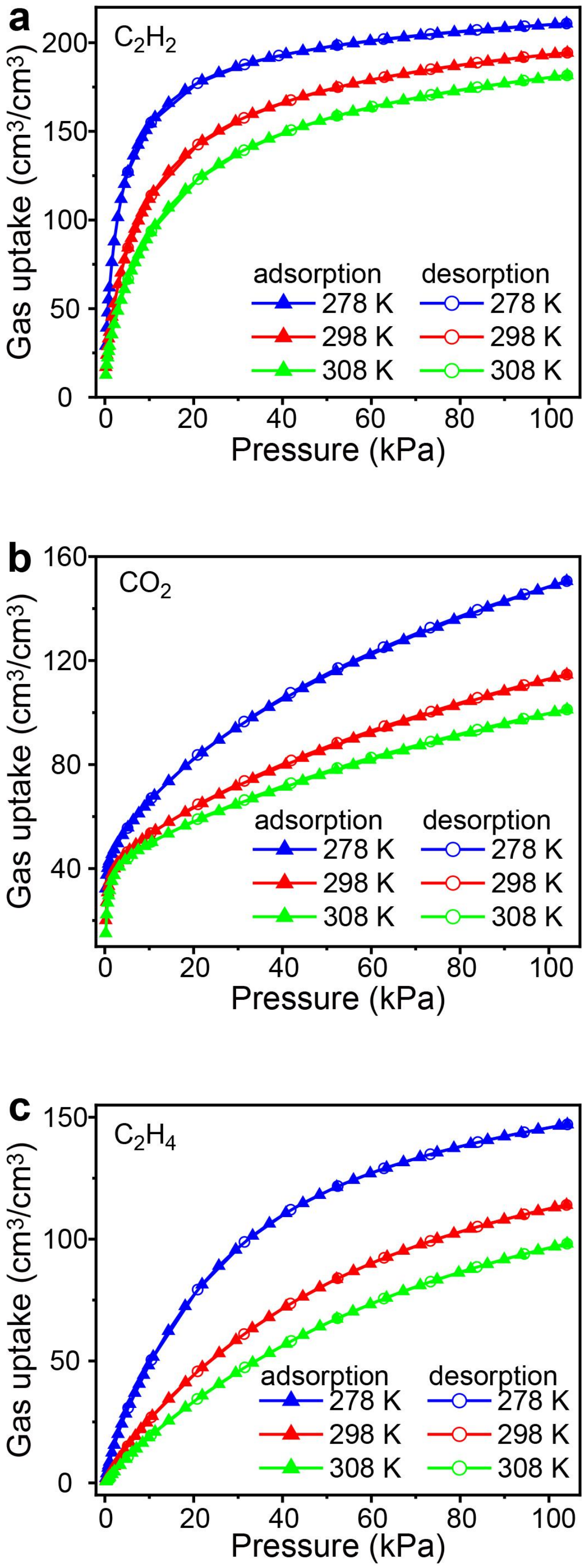

Supplementary Figure 12. The adsorption and desorption isotherms of C_2H_2 (a), CO_2(b), and C_2H_4 (c) on **ZNU-6** at 278, 298, and 308 K.

Supplementary Table 3. Dual-site Langmuir parameter fits for C_2H_2, CO_2 adsorption isotherms and single-site Langmuir parameter fit for C_2H_4 adsorption isotherms in ZNU-6.

	Site A			Site B		
	$q_{A,sat}$ mol/kg	b_{A0} Pa^{-1}	E_A kJ mol^{-1}	$q_{B,sat}$ mol/kg	b_{B0} Pa^{-1}	E_B kJ mol^{-1}
C_2H_2	1.2	1.067E-09	37.5	7.6	5.015E-11	35.4
CO_2	7.7	2.01E-10	25.5	1.8	1.06E-10	37.2
C_2H_4	7.6	1.339E-10	29			

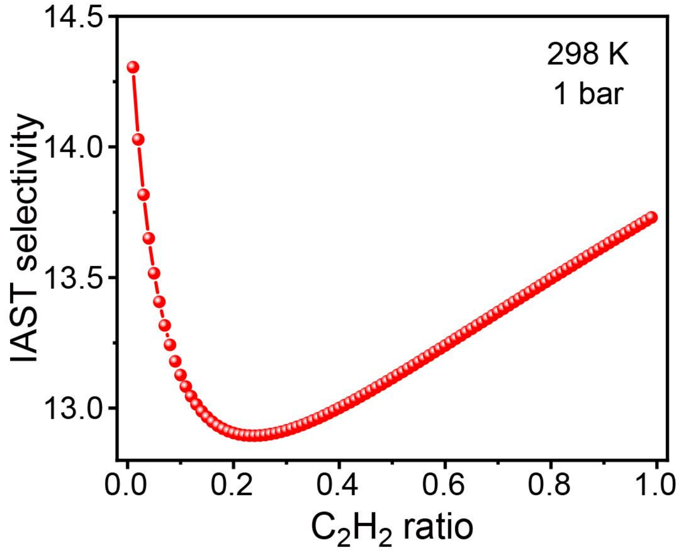

Supplementary Figure 13. IAST selectivity of **ZNU-6** towards gas mixtures of C_2H_2/C_2H_4 with different ratios at 298 K and 1 bar.

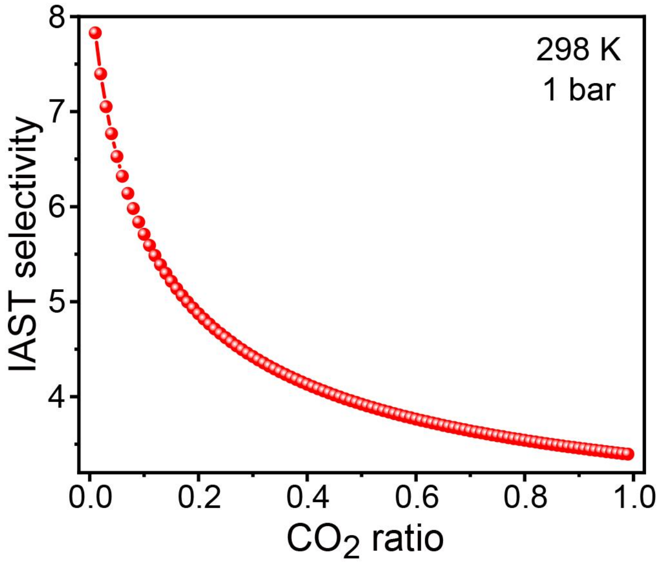

Supplementary Figure 14. IAST selectivity of **ZNU-6** towards gas mixtures of CO_2/C_2H_4 with different ratios at 298 K and 1 bar.

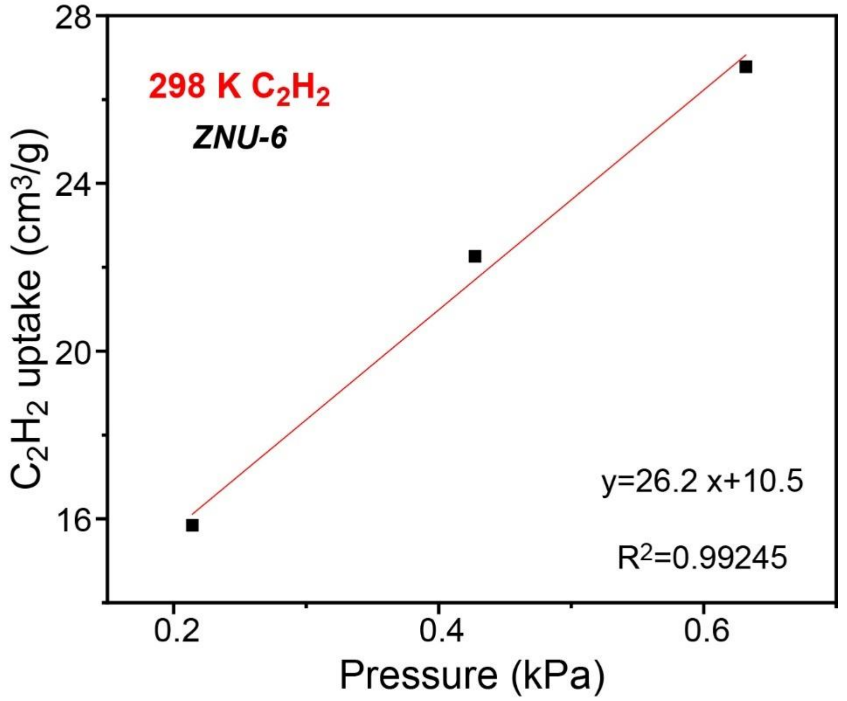

Supplementary Figure 15.. C_2H_2 adsorption isotherm of **ZNU-6** at 298 K fitted with Henry's equation at low pressure (0.2 kPa-0.7 kPa).

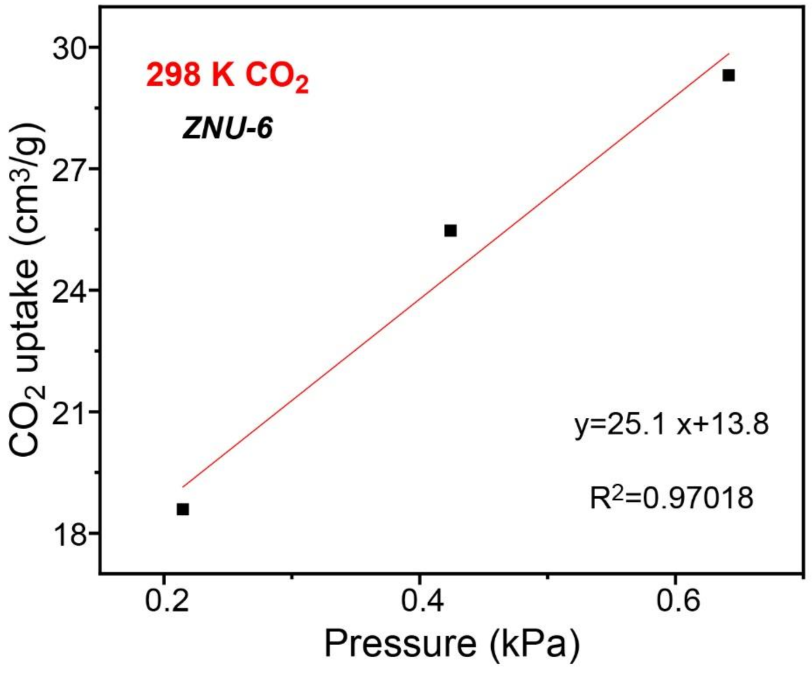

Supplementary Figure 16. CO_2 adsorption isotherm of **ZNU-6** at 298 K fitted with Henry's equation at low pressure (0.2 kPa-0.7 kPa).

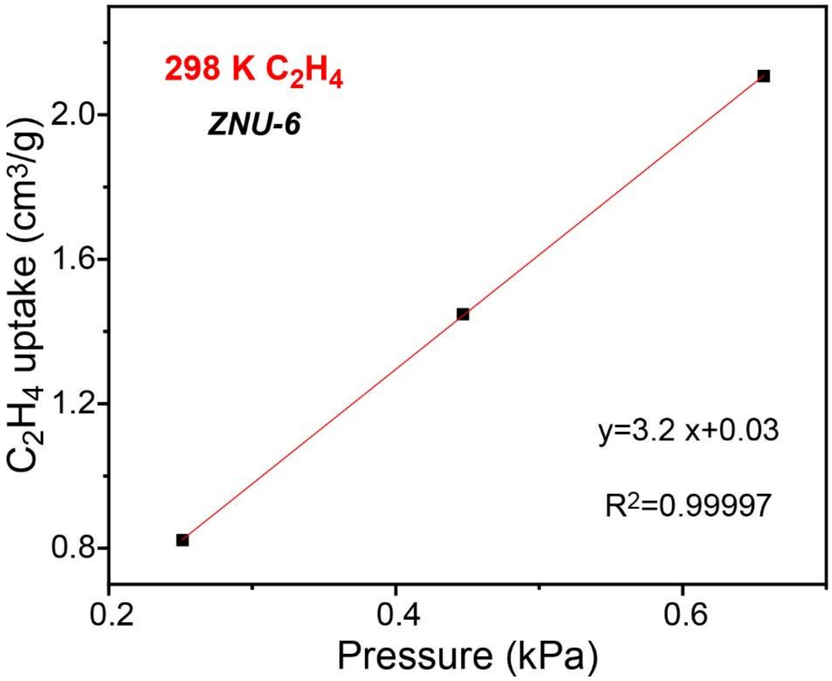

Supplementary Figure 17. C_2H_4 adsorption isotherm of **ZNU-6** at 298 K fitted with Henry's equation at low pressure (0.2 kPa-0.7 kPa).

Supplementary Table 4. Summary of Henry constant and Henry's selectivity of ZNU-6

	Henry constant (cm^3 g^{-1} kPa^{-1})
C_2H_2	26.2
CO_2	25.1
C_2H_4	3.2
**C_2H_2/C_2H_4 Henry's selectivity**	**8.2**
**CO_2/C_2H_4 Henry's selectivity**	**7.8**

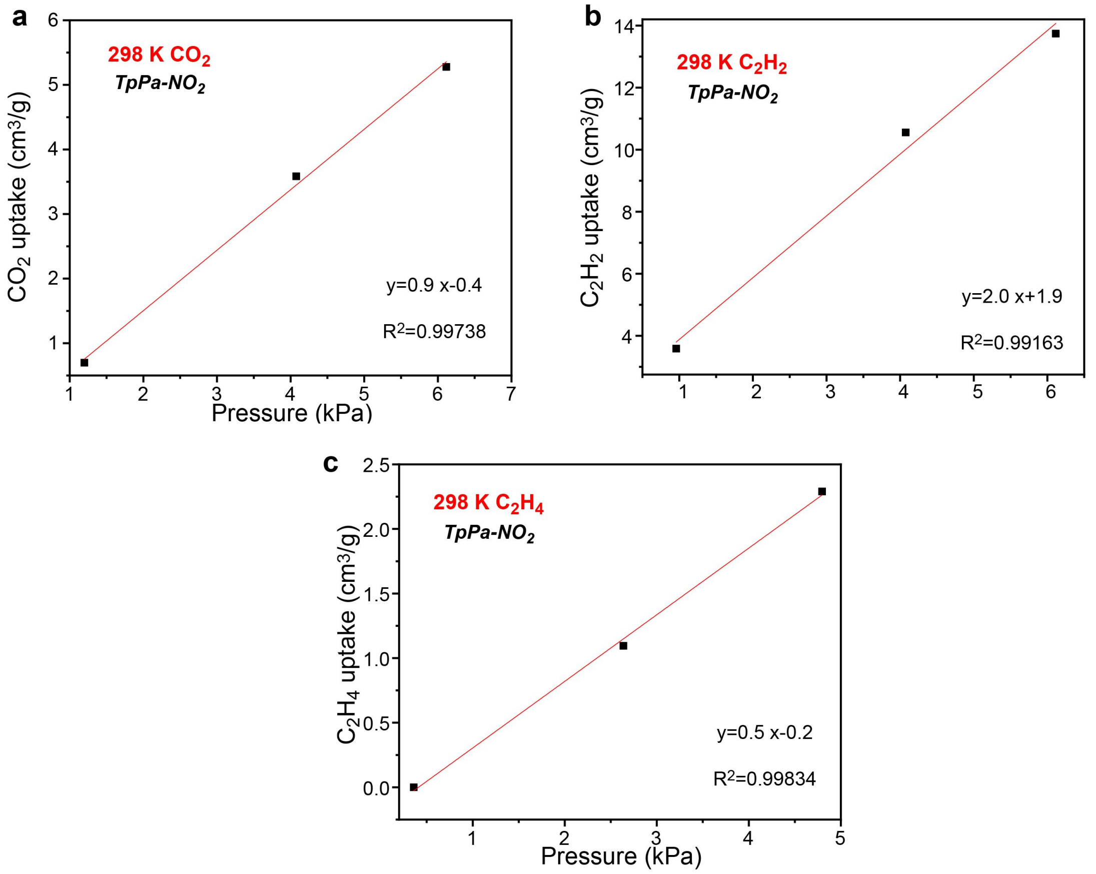

Supplementary Figure 18. $CO_2/C_2H_2/C_2H_4$ adsorption isotherm of **TpPa-NO$_2$** at 298 K fitted with Henry's equation at low pressure (0-7 kPa). Data of adsorption was from Reference 1.

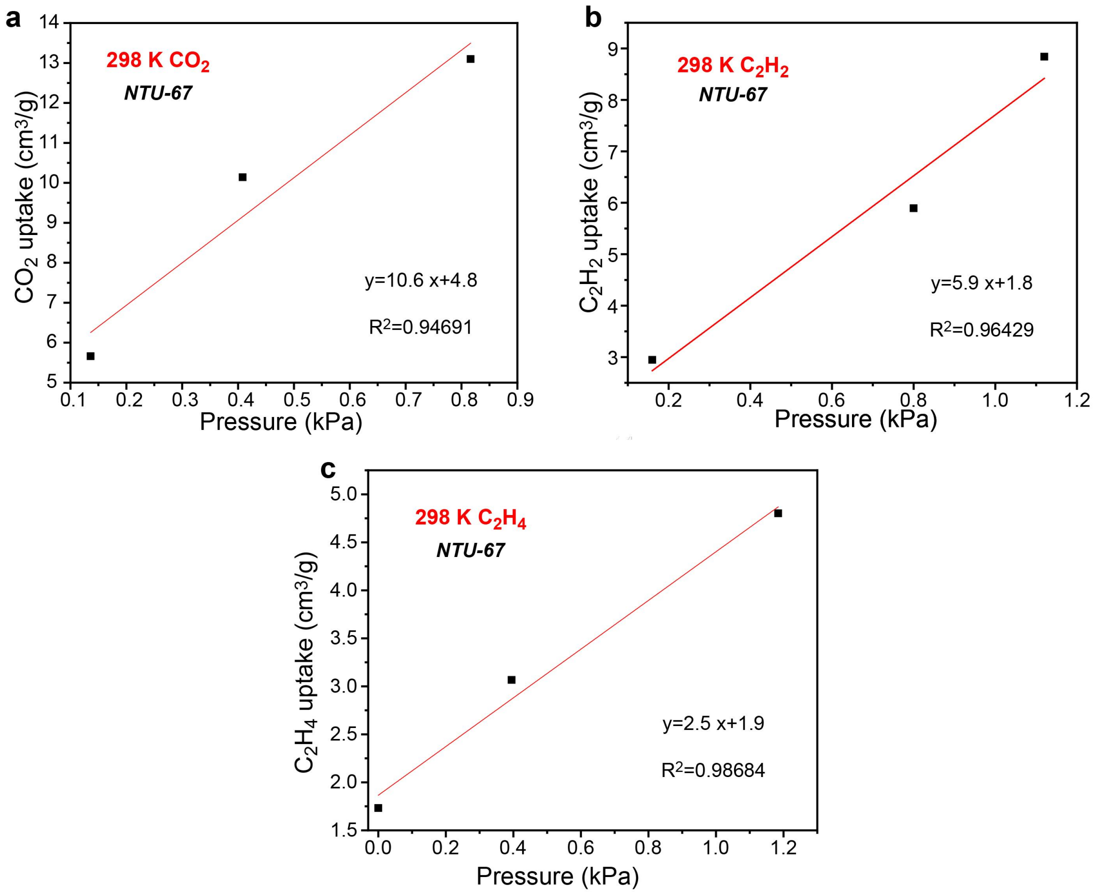

Supplementary Figure 19. CO_2/C_2H_2/C_2H_4 adsorption isotherm of **NTU-67** at 298 K fitted with Henry's equation at low pressure (0-1.2 kPa). Data of adsorption was from Reference 2.

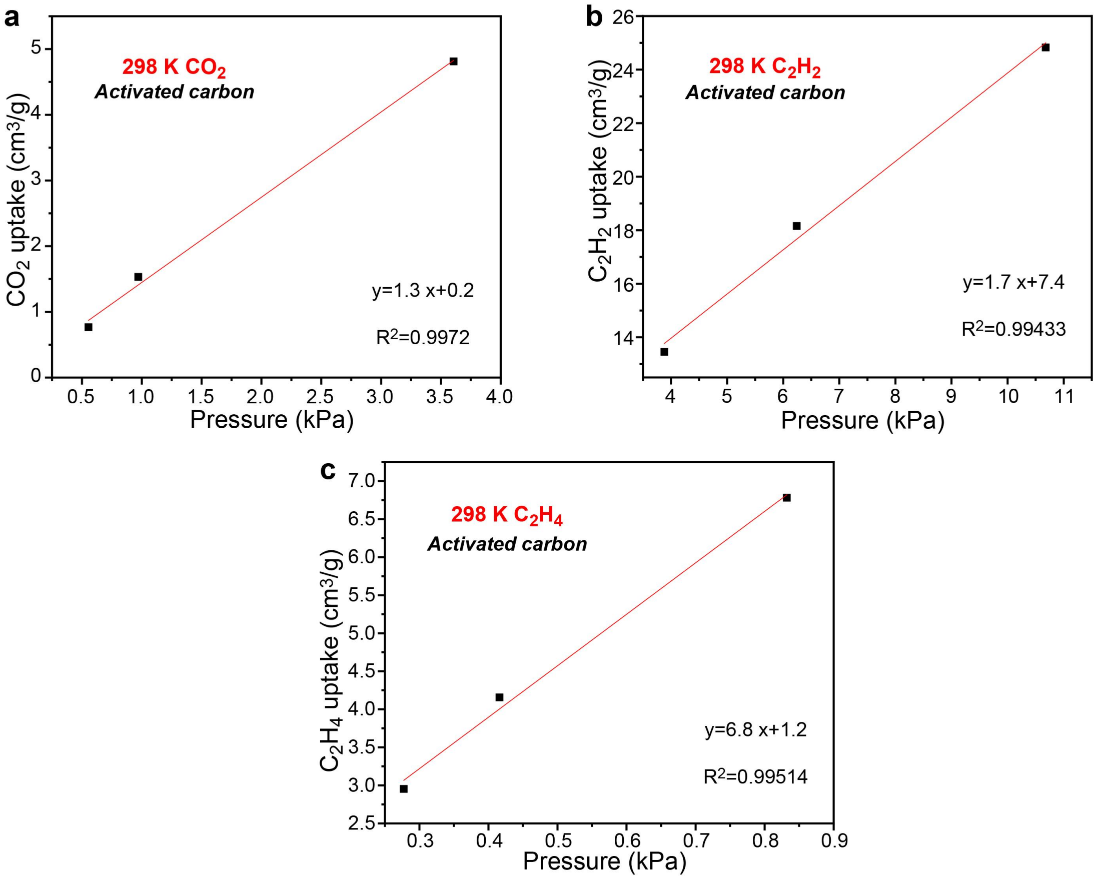

Supplementary Figure 20. CO_2/C_2H_2/C_2H_4 adsorption isotherm of activated carbon at 298 K fitted with Henry's equation at low pressure (0-11.0 kPa). Data of adsorption was from Reference 2.

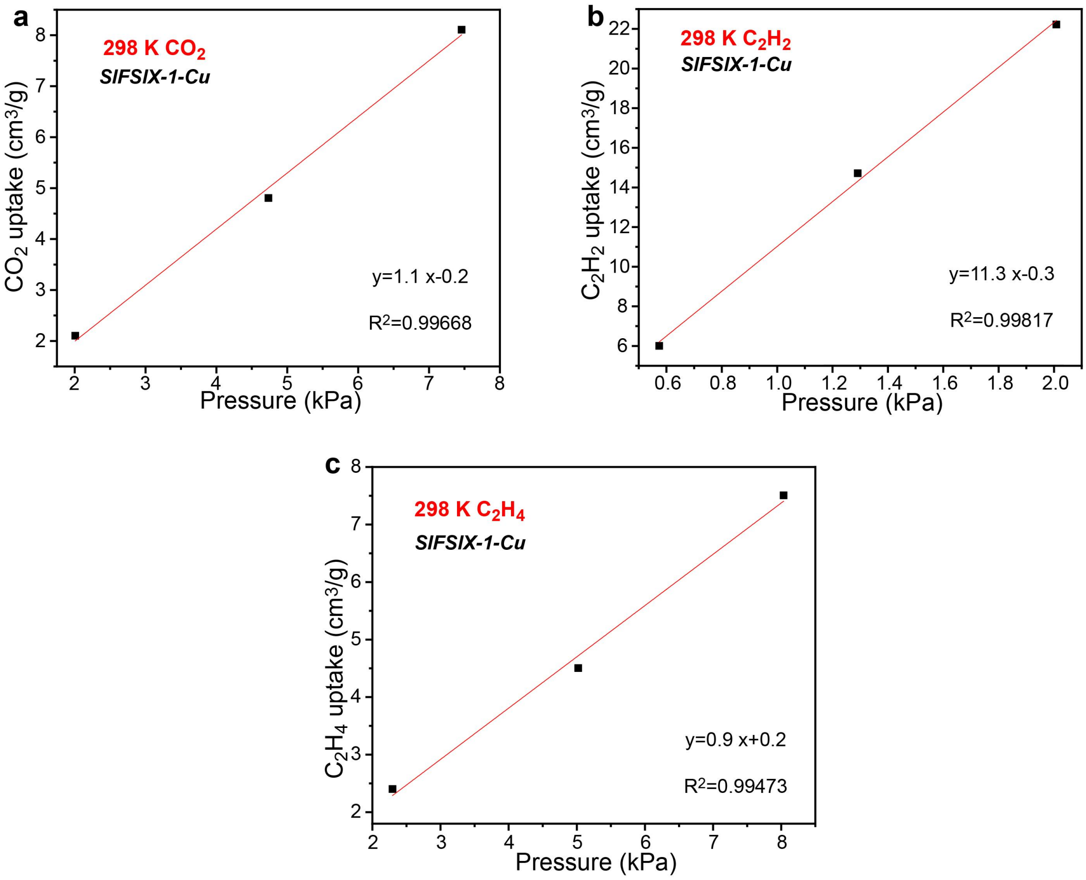

Supplementary Figure 21. $CO_2/C_2H_2/C_2H_4$ adsorption isotherm of SIFSIX-1-Cu at 298 K fitted with Henry's equation at low pressure (0-8.0 kPa). Data of adsorption was from Reference 2.

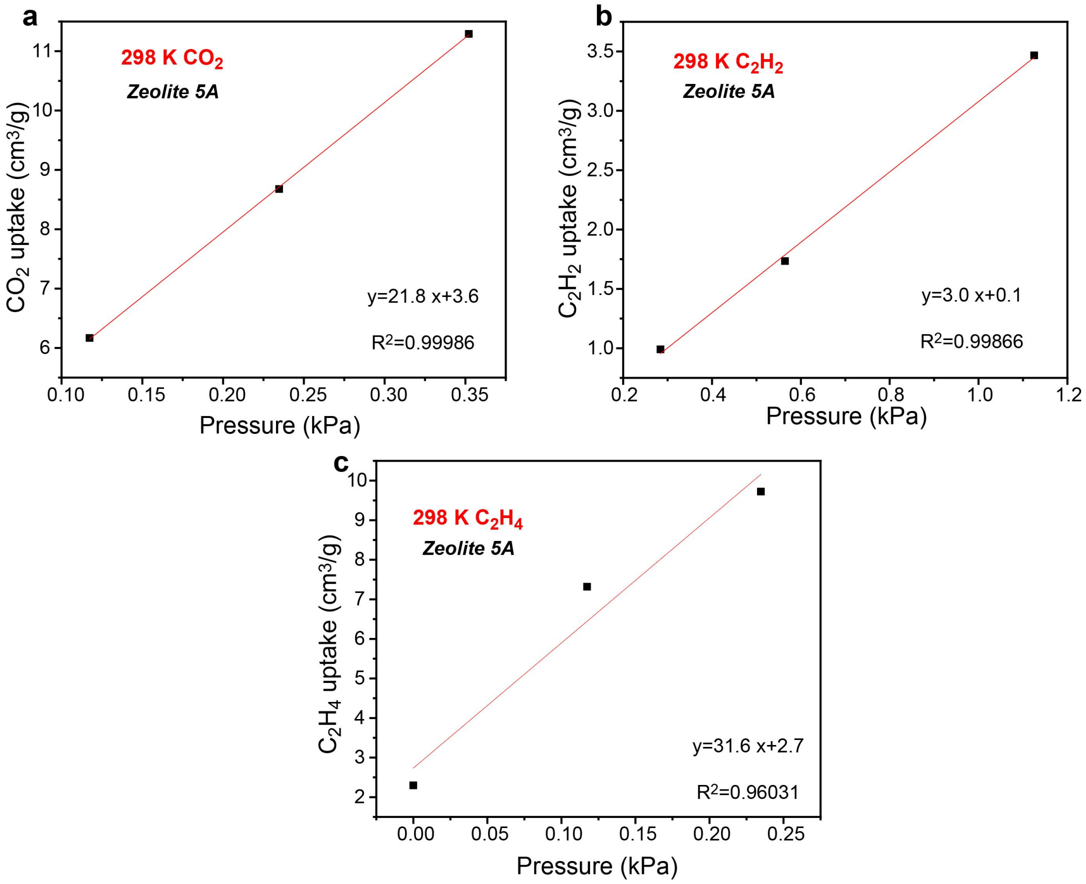

Supplementary Figure 22. $CO_2/C_2H_2/C_2H_4$ adsorption isotherm of Zeolite 5A at 298 K fitted with Henry's equation at low pressure (0-1.2 kPa). Data of adsorption was from Reference 2.

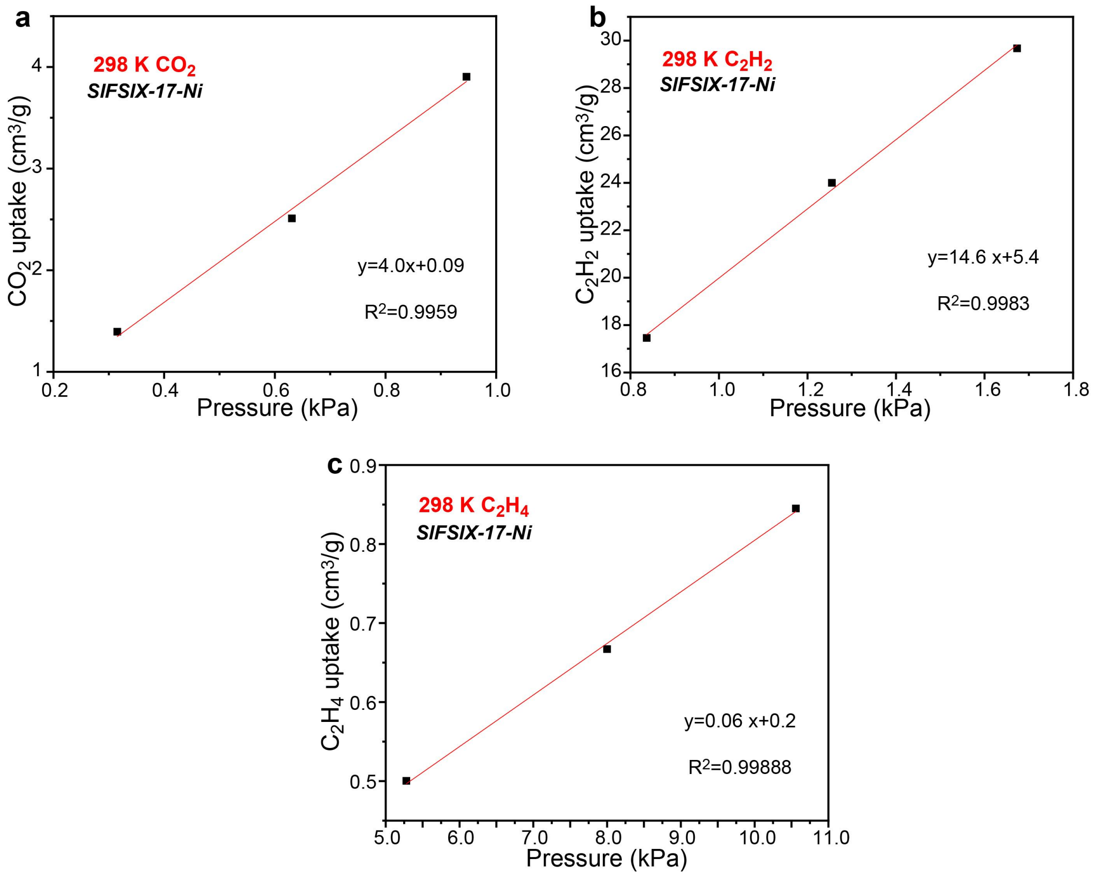

Supplementary Figure 23. CO_2/C_2H_2/C_2H_4 adsorption isotherm of SIFSIX-17-Ni at 298 K fitted with Henry's equation at low pressure (0-1.8 kPa). Data of adsorption was from Reference 3.

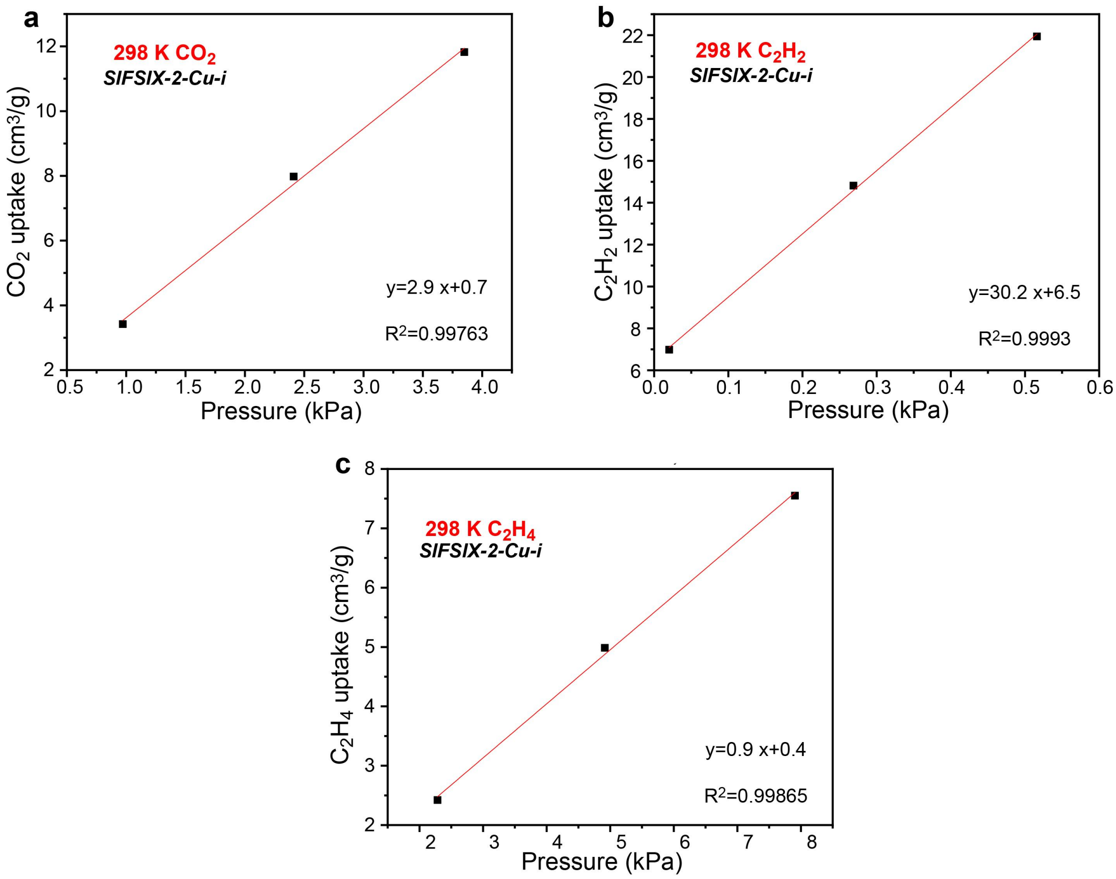

Supplementary Figure 24. CO_2/C_2H_2/C_2H_4 adsorption isotherm of SIFSIX-2-Cu-i at 298 K fitted with Henry's equation at low pressure (0-0.6 kPa). Data of adsorption was from Reference 2.

Supplementary Table 5. Comparison of Henry constants and Henry's selectivity among absorbents used in single-step C_2H_4 purification from ternary gas mixture.

	C_2H_2 Henry constant (cm^3 g^{-1} kPa^{-1})	CO_2 Henry constant (cm^3 g^{-1} kPa^{-1})	C_2H_4 Henry constant (cm^3 g^{-1} kPa^{-1})	C_2H_2/C_2H_4 Henry's selectivity	CO_2/C_2H_4 Henry's selectivity
TpPa-NO_2	2.0	0.9	0.5	4.0	1.8
NTU-67	5.9	10.6	2.5	2.36	4.24
Activated carbon	1.7	1.3	6.8	0.25	0.19
SIFSIX-1-Cu	11.3	1.1	0.9	12.56	1.22
Zeolite 5A	3.0	21.8	31.6	0.09	0.69
SIFSIX-17-Ni	14.6	4.0	0.06	243.33	66.67
SIFSIX-2-Cu-i	30.2	2.9	0.9	33.56	3.22
**ZNU-6**	**26.2**	**25.1**	**3.2**	**8.19**	**7.84**

Supplementary Table 6. Comparison of saturated C_2H_2 and CO_2 uptake (298 K, 1 bar).

	C_2H_2 uptake cm^3/g (cm^3/cm^3)	CO_2 uptake cm^3/g (cm^3/cm^3)	Ref
TIFSIX-3-Ni	67.2	52.0	3
SIFSIX-3-Ni	73.9	60.5	3-5
SIFSIX-17-Ni	73.9	51.5	3
SIFSIX-3-Zn	81.5	57.1	4-6
TIFSIX-17-Ni	73.9	49.3	3
SIFSIX-3-Cu	83.5	56.0	4, 6
UTSA-300a	68.9	3.3	7
NTU-67	73.7	45.7	2
NTU-65	75.4	2.3	8
UTSA-200a	81.8	105.5	9, 10
SIFSIX-2-Cu-i	89.6	108.6	4, 10, 11
ZU-62	82.2	/	12
ZNU-4	85.1	44.1	13
NCU-100a	102.3	13.7	14
ZNU-5	128.6	15.2	15
SIFSIX-1-Cu (ρ=0.864 g/cm^3)	190.4 (164.5)	117.6 (101.6)	4
SIFSIX-Cu-TPA (ρ=0.995 g/cm^3)	185.0 (184.1)	107.0 (106.5)	16
**ZNU-6**	**180.44 (193.8)**	**106.7 (114.6)**	**This work**

Supplementary Table 7. Comparison of C_2H_2 and CO_2 uptake per anion among anion pillared MOFs.

	Molecule formula	Molecular weight	C_2H_2/anion ratio	CO_2/anion ratio	Ref.
TIFSIX-3-Ni	$Ni(TiF_6)(C_4H_4N_2)_2$	379.95	1.14	0.88	3
SIFSIX-3-Ni	$Ni(SiF_6)(C_4H_4N_2)_2$	360.96	1.19	0.97	3-5
SIFSIX-17-Ni	$Ni(SiF_6)(C_4H_5N_3)_2$	390.98	1.29	0.9	3
SIFSIX-3-Zn	$Zn(SiF_6)(C_4H_4N_2)_2$	367.64	1.34	0.93	4-6
TIFSIX-17-Ni	$Ni(TiF_6)(C_4H_5N_3)_2$	410.76	1.36	0.91	3
SIFSIX-3-Cu	$Cu(SiF_6)(C_4H_4N_2)_2$	365.80	1.37	0.92	4,6
UTSA-300a	$Zn(SiF_6)(C_{10}H_8SN_2)_2$	583.95	1.8	0.09	7
NTU-67	$Cu(SiF_6)(C_{12}H_{10}N_4)_2$	626.12	2.06	1.28	2
NTU-65	$Cu(SiF_6)(C_{12}H_{10}N_4)_2$	626.12	2.11	0.06	8
UTSA-200a	$Cu(SiF_6)(C_{10}H_8N_4)_2$	574.04	2.10	2.71	9-10
SIFSIX-2-Cu-i	$Cu(SiF_6)(C_{12}H_8N_2)_2$	565.78	2.27	3.06	4,10-11
ZU-62	$Cu(NbOF_5)(C_{12}H_8N_2)_2$	627.86	2.31	/	12
ZNU-4	$Cu(TiF_6)(C_{12}H_{10}N_4)_2$	645.88	2.45	1.27	13
NCU-100a	$Cu(SiF_6)(C_{10}H_{14}N_2O_3S)_2$	690.21	3.15	0.42	14
ZNU-5	$Cu(TiF_6)(C_{12}H_{10}N_4)_2$	645.88	3.71	0.44	15
SIFSIX-1-Cu	$Cu(SiF_6)(C_{10}H_8N_2)_2$	517.78	4.40	2.72	4
SIFSIX-Cu-TPA	$Cu(SiF_6)(C_{15}H_{12}N_4)_{1.33}$	536.67	4.44	2.57	16
**ZNU-6**	**$Cu(GeF_6)(C_{15}H_{12}N_4)_{1.33}$**	**581.21**	**4.68**	**2.77**	**This work**

Guest/anion ratio=$Q_{gas} \times M \div 22.4 \div 1000$

Qgas: the gas uptake of APMOFs, mL/g

M: Molecular weight of crystals, g/mol

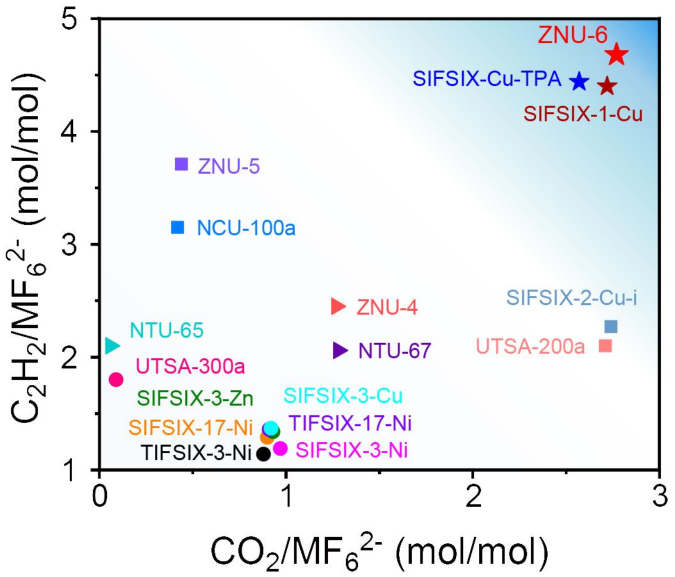

Supplementary Figure 25. Comparison of the saturated C_2H_2 and CO_2 uptake (1 bar, 298 K) among anion pillared MOFs.

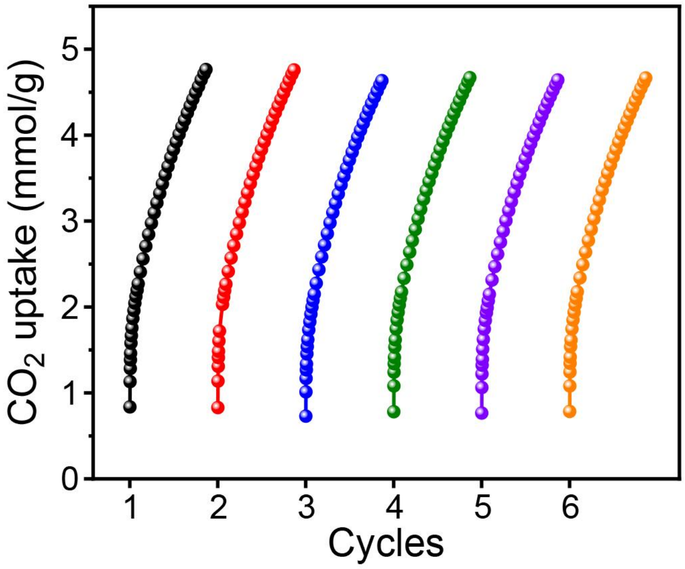

Supplementary Figure 26. Six cycles of CO_2 adsorption of **ZNU-6** at 298 K.

The reactivation condition between 5th and 6th cycle: Under vacuum, at room temperature for 3 hours.

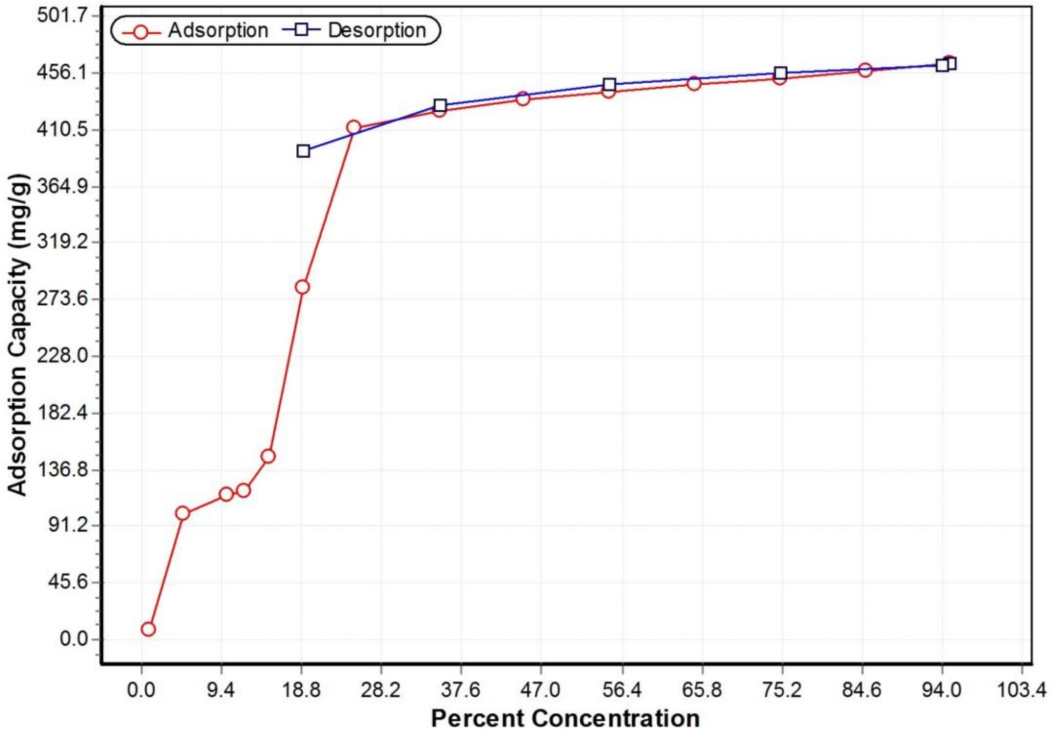

Supplementary Figure 27. H_2O adsorption isotherms of **ZNU-6** at 298 K.

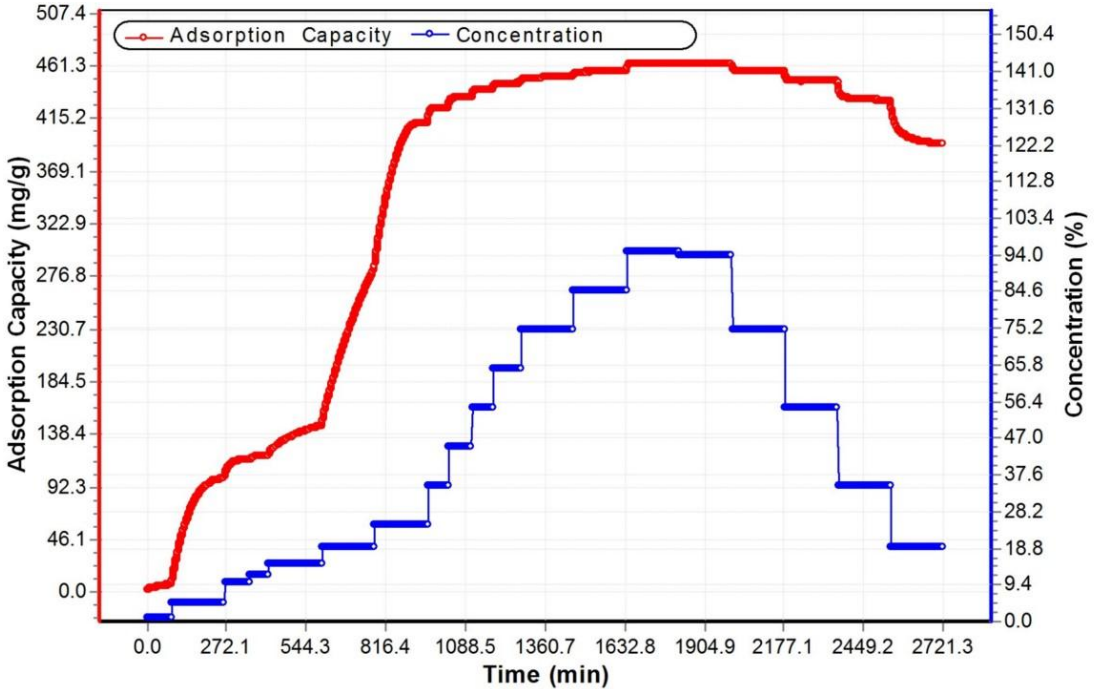

Supplementary Figure 28. H_2O adsorption capacity-time curve (adsorption kinetics) of **ZNU-6** at 298 K.

IV Kinetic studies

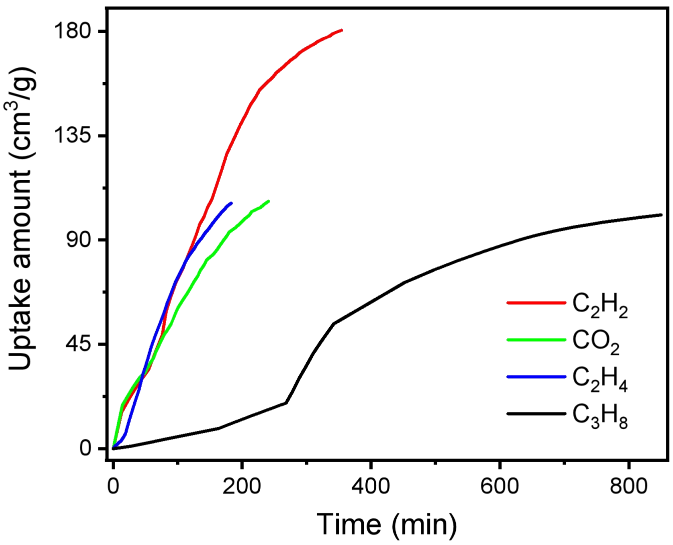

Supplementary Figure 29. Time-dependent adsorption curves of C_2H_2, CO_2, C_2H_4 and C_3H_8.

Analysis: Despite the narrow channel size in ZNU-6, the diffusion of C_2H_2, CO_2 and C_2H_4 are very fast. The measurements are finished within 350 min to reach saturated uptake. When compared, C_3H_8 with larger molecular size is less kinetic-favoured, the measurement takes over 800 min under the same conditions.

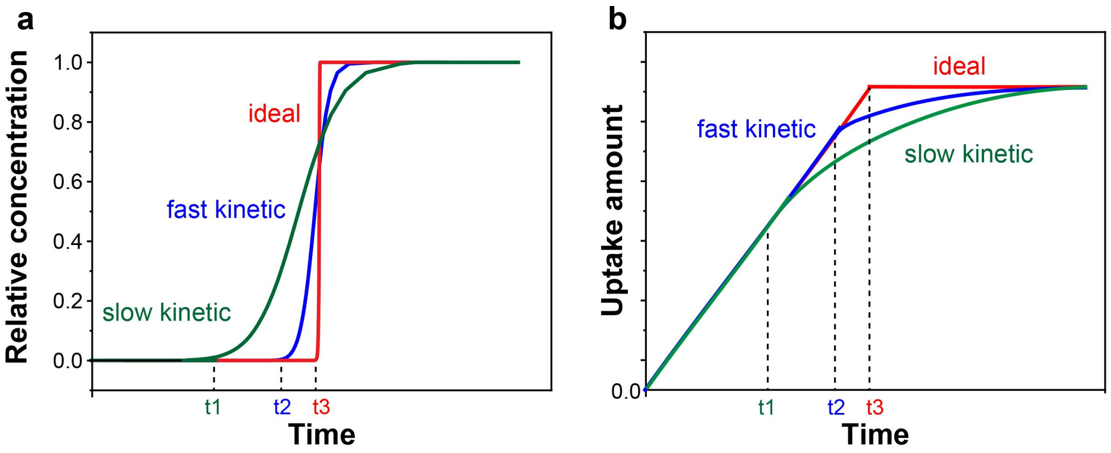

Supplementary Figure 30. Typical breakthrough curves (a) and the calculated kinetic curves therefrom.

1. Ideal materials (red curves) feature nearly vertical breakthrough curves, which is almost impossible to realize in real system due to the lateral diffusion as well as the diffusion between the particles in the fixed bed.

2. Materials with fast kinetic (blue curves) feature very sharp breakthrough curves, indicating the gas diffusion within the pores are very fast. The breakthrough phenomenon occurs when the material is nearly get saturated.

3. Materials with slow kinetic (green curves) feature relatively flat breakthrough curves. Due to the slow diffusion within the pores, tested gas flows through the space between particles instead of diffusion into the pores to be captured. Thus, the breakthrough phenomenon occurs when the material is far from gas-saturation.

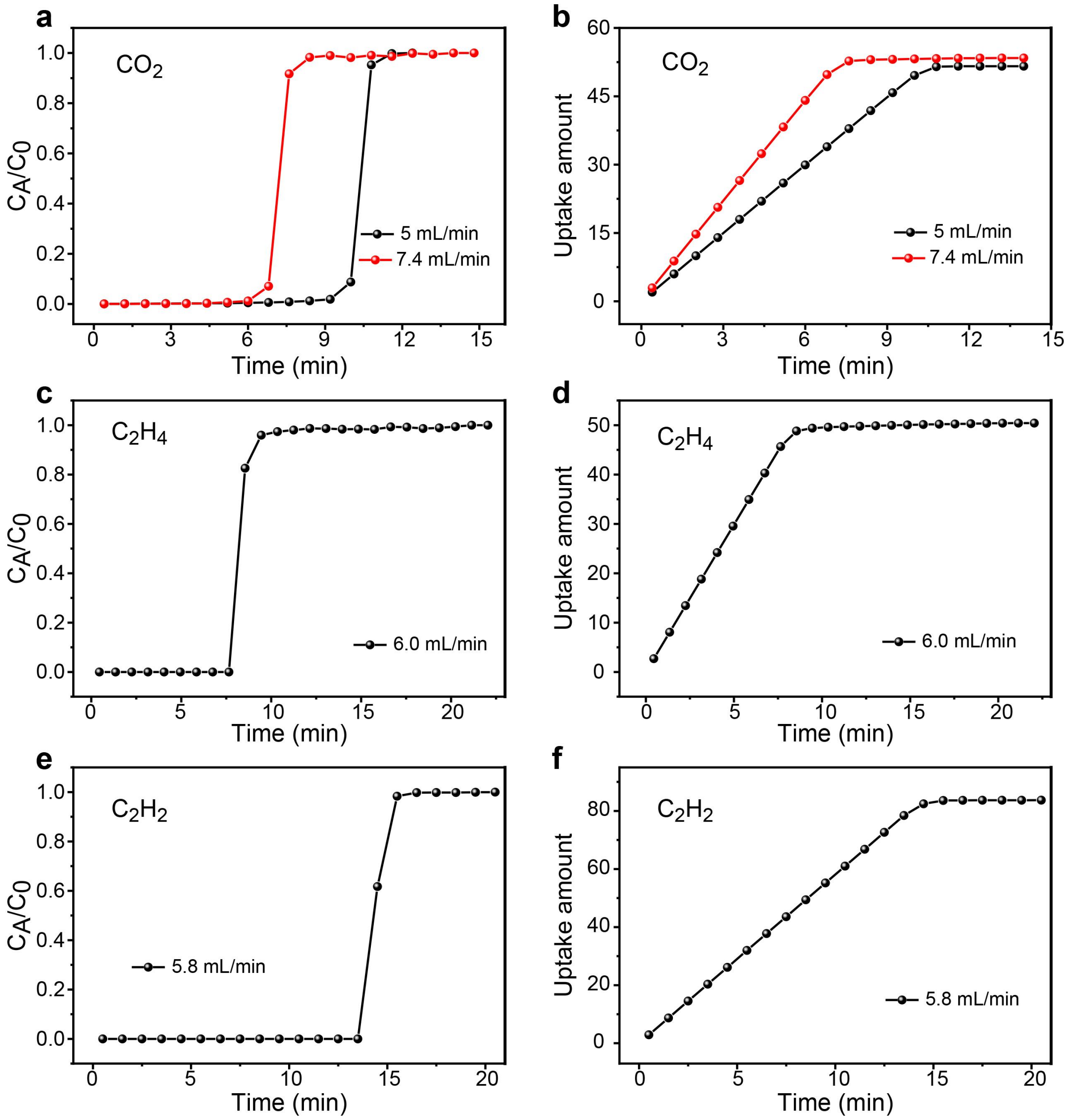

Supplementary Figure 31. Experimental breakthrough curves of pure gases in ZNU-6 and the calculated kinetic curves therefrom (a, b) CO_2; (c, d) C_2H_4; (e, f) C_2H_2.

The breakthrough curves of C_2H_2, CO_2 and C_2H_4 increase sharply after the gas break out, and the corresponding kinetic curves rises rapidly, indicating that ZNU-6 has fast kinetic for CO_2, C_2H_4 and C_2H_2 adsorption. Notably, the flow rate of > 5mL/min is relatively fast for breakthrough experiments; most breakthrough experiments reported in published papers were performed at ~ 2 mL/min. Thus, the retained sharp breakthrough curves under such high flowrate fully confirm the fast diffusion of CO_2, C_2H_4 and C_2H_2 in the pores of ZNU-6, which is very advantageous for practical applications

Experimental method: The column packed with ZNU-6 was activated completely firstly, and then pure CO_2, C_2H_4 or C_2H_2 was introduced at a specific flow rate. The measure range of our flowmetre is 0-10 sccm. The real flowrate is calibrated by self-made soapfilm flowmetre. The displayed and real flowrate is shown in Supplementary Table S8.

Supplementary Table 8. Comparison of the displayed and calibrated actual flowrate.

	Displayed flow rate (mL/min)	Actual flow rate (mL/min)
CO_2	6.8	5.0
CO_2	10.0	7.4
C_2H_4	10.0	6.0
C_2H_2	10.0	5.8

V Breakthrough experiments

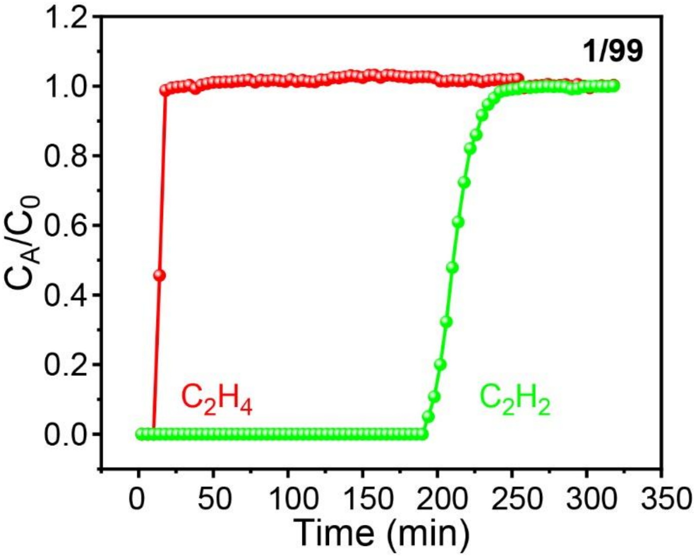

Supplementary Figure 32. Experimental breakthrough curves of **ZNU-6** for C_2H_4/C_2H_2 (1/99).

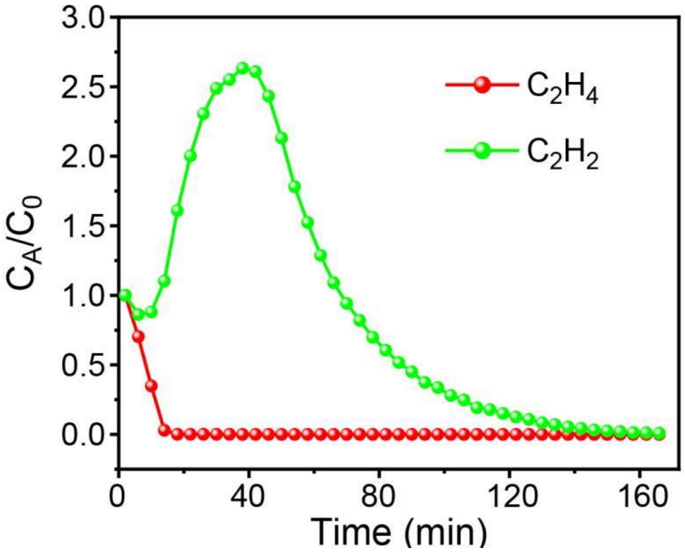

Supplementary Figure 33. Experimental dynamic desorption curves of **ZNU-6** after breakthrough experiment of C_2H_2/C_2H_4 (1/99). Desorption conditions: Ar flow rate 10 mL/min at 75 °C.

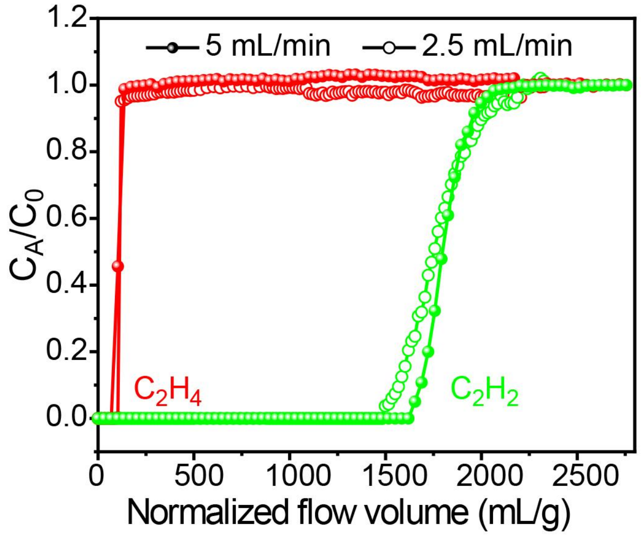

Supplementary Figure 34. Experimental breakthrough curves of **ZNU-6** for C_2H_2 /C_2H_4 (1/99) at different flow rate.

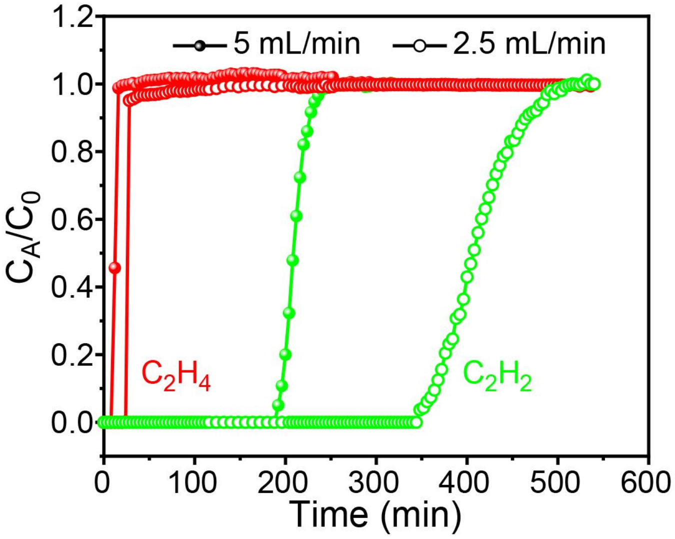

Supplementary Figure 35. Experimental breakthrough curves of **ZNU-6** for C_2H_2/C_2H_4 (1/99) at different flow rate.

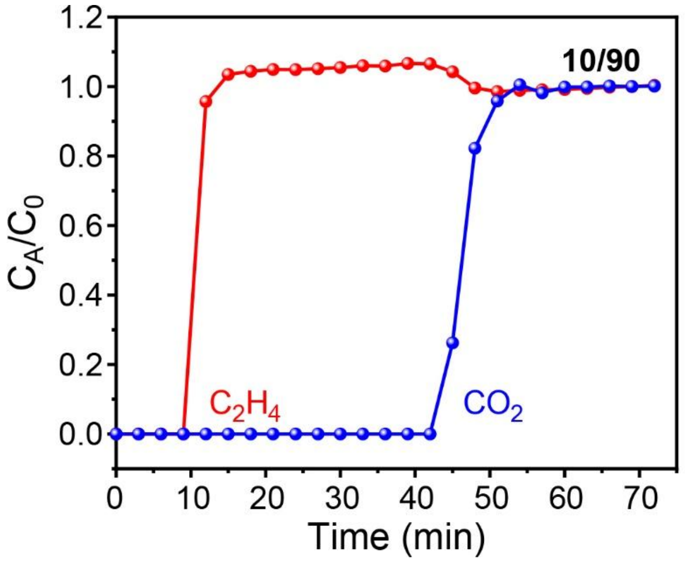

Supplementary Figure 36. Experimental breakthrough curves of **ZNU-6** for CO_2/C_2H_4 (10/90).

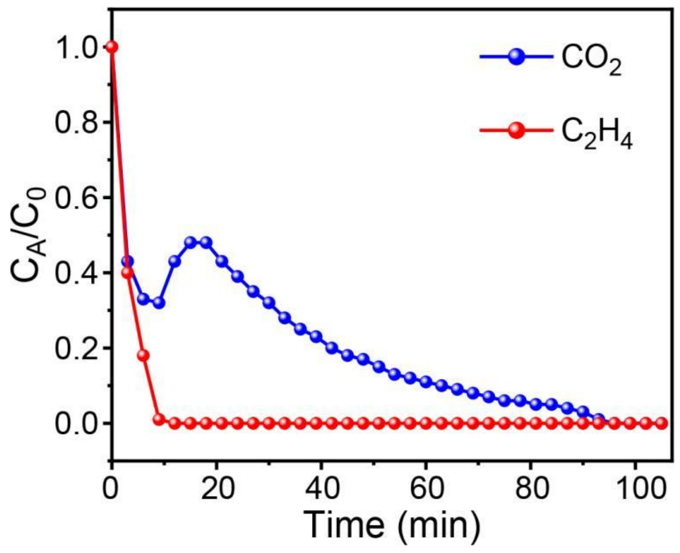

Supplementary Figure 37. Experimental dynamic desorption curves of **ZNU-6** after breakthrough experiment of CO_2/C_2H_4 (10/90). Desorption conditions: Ar flow rate 10 mL/min at 75 °C.

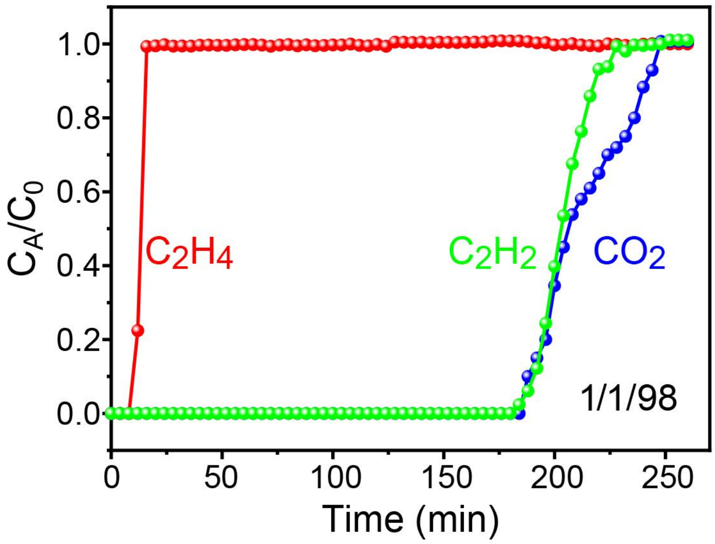

Supplementary Figure 38. Experimental breakthrough curves of **ZNU-6** for $C_2H_2/CO_2/C_2H_4$ (1/1/98). Flow rate: 5 mL/min.

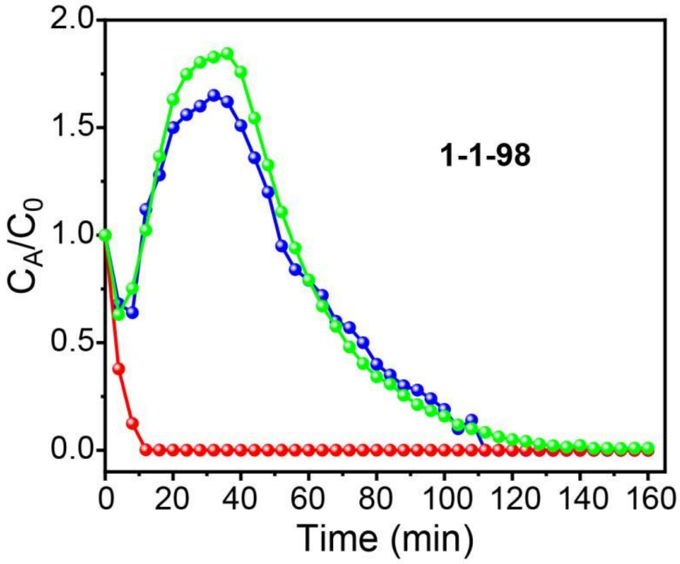

Supplementary Figure 39. Experimental dynamic desorption curves of **ZNU-6** after breakthrough experiment of $C_2H_2/CO_2/C_2H_4$ (1/1/98). Desorption conditions: Ar flow rate 10 mL/min at 75 °C.

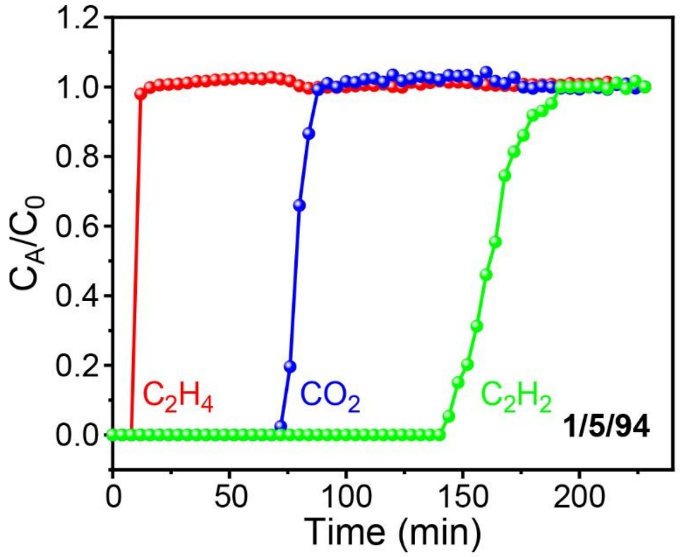

Supplementary Figure 40. Experimental breakthrough curves of **ZNU-6** for $C_2H_2/CO_2/C_2H_4$ (1/5/94). Flow rate: 5 mL/min.

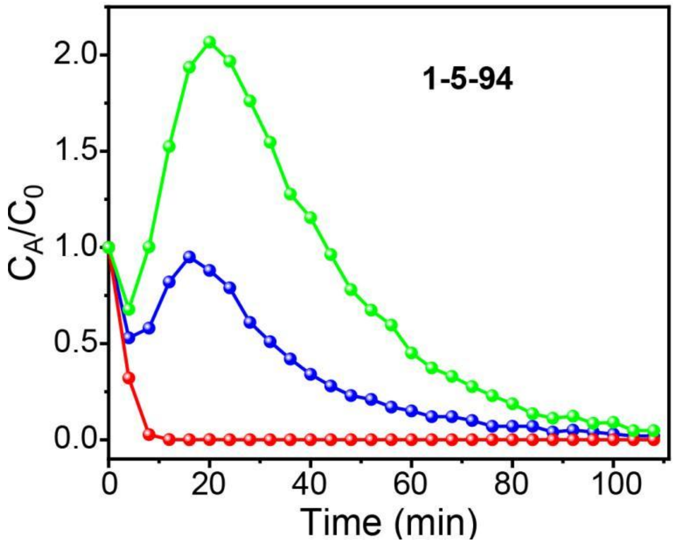

Supplementary Figure 41. Experimental dynamic desorption curves of **ZNU-6** after breakthrough experiment of $C_2H_2/CO_2/C_2H_4$ (1/5/94). Desorption conditions: Ar flow rate 10 mL/min at 75 °C.

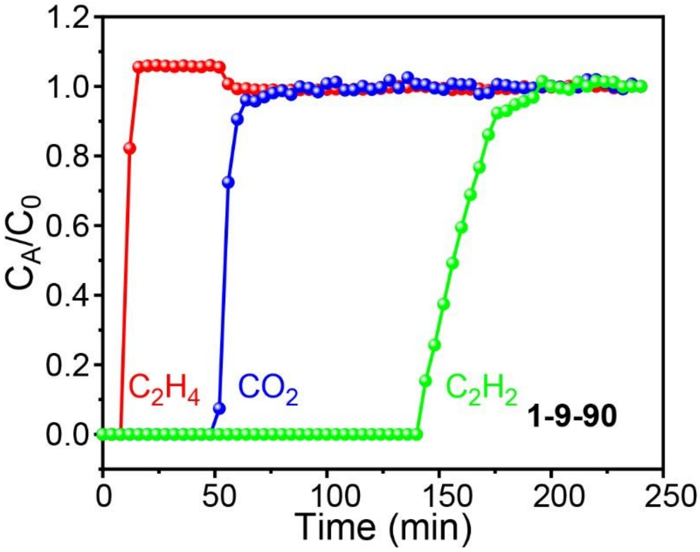

Supplementary Figure 42. Experimental breakthrough curves of **ZNU-6** for $C_2H_2/CO_2/C_2H_4$ (1/9/90). Flow rate: 5 mL/min.

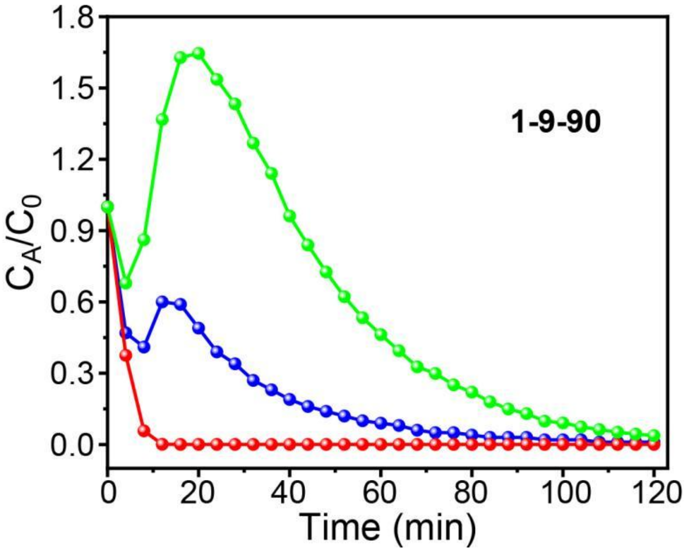

Supplementary Figure 43. Experimental dynamic desorption curves of **ZNU-6** after breakthrough experiment of $C_2H_2/CO_2/C_2H_4$ (1/9/90). Desorption conditions: Ar flow rate 10 mL/min at 75 °C.

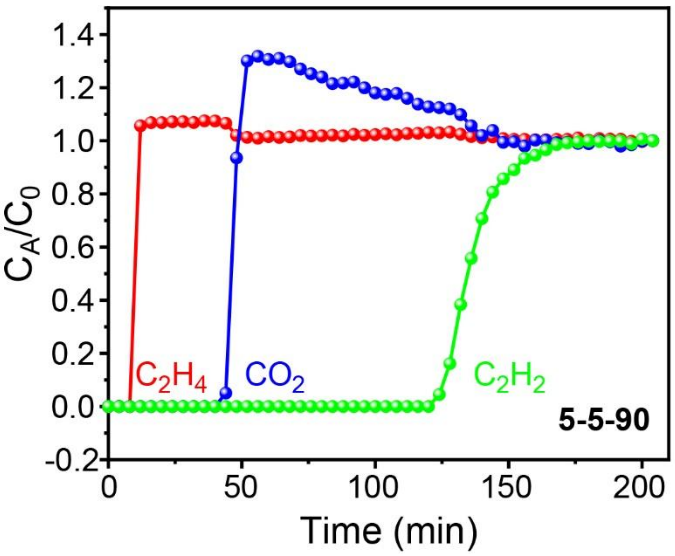

Supplementary Figure 44. Experimental breakthrough curves of **ZNU-6** for $C_2H_2/CO_2/C_2H_4$ (5/5/90). Flow rate: 5 mL/min.

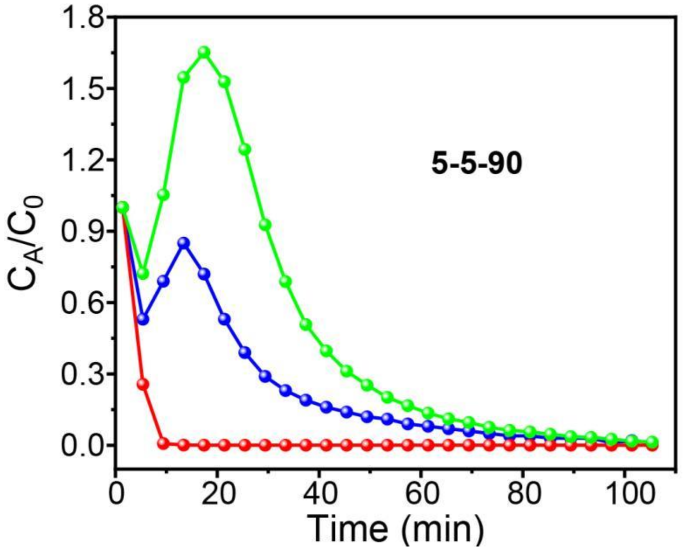

Supplementary Figure 45. Experimental dynamic desorption curves of **ZNU-6** after breakthrough experiment of $C_2H_2/CO_2/C_2H_4$ (5/5/90). Desorption conditions: Ar flow rate 10 mL/min at 75 °C.

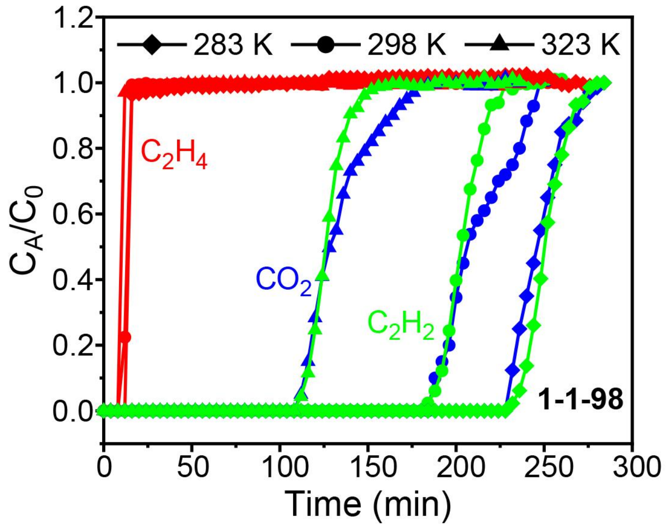

Supplementary Figure 46. Experimental breakthrough curves of **ZNU-6** for $C_2H_2/CO_2/C_2H_4$ (1/1/98) at 283, 298 and 323 K. Flow rate: 5 mL/min.

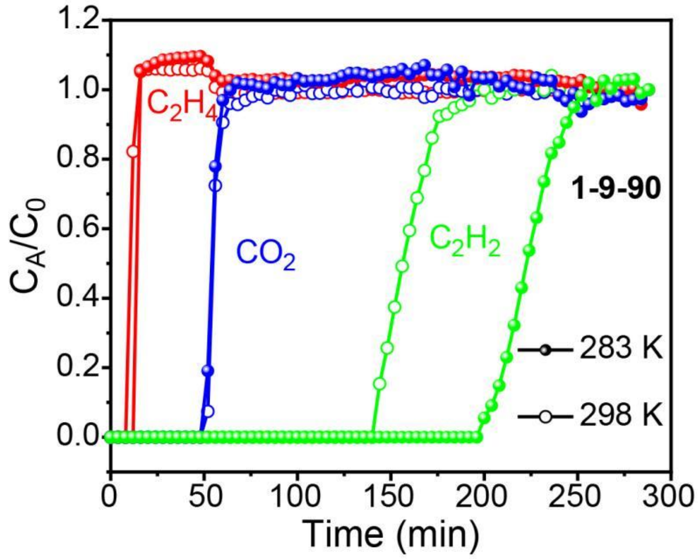

Supplementary Figure 47. Experimental breakthrough curves of **ZNU-6** for $C_2H_2/CO_2/C_2H_4$ (1/9/90) at 283 and 298 K. Flow rate: 5 mL/min.

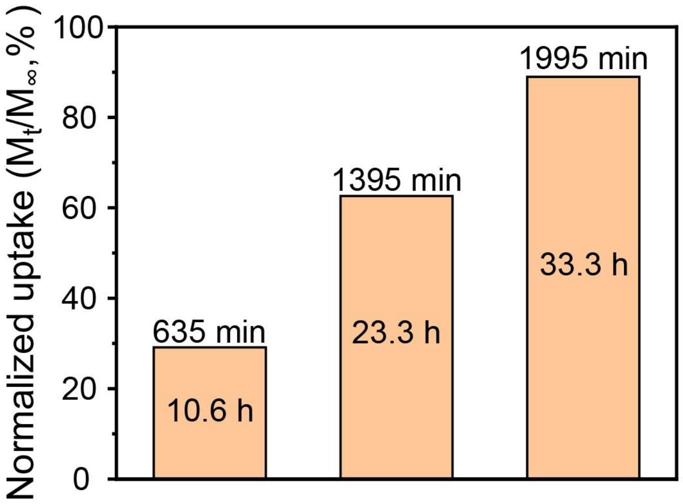

Supplementary Figure 48. H_2O uptake in breakthrough experiments (N_2, RH=100%). Flow rate: 5 mL/min.

Experimental method: The column packed with ZNU-6 was activated completely firstly, and then N_2 with saturated moisture was introduced at a flow rate of 5 mL/min. After each period, the column was picked out and weighted by balance to calculate the adsorbed amount of water. The adsorbed N_2 amount is neglected.

Supplementary Table 9. Experimental dynamic C_2H_4 productivity and captured C_2H_2/CO_2 amount for ZNU-6 from different gas ratios and under different conditions.

**Conditions**	**Experimental C_2H_4 productivity (mol/kg)**	**Experimental C_2H_2 captured amount (mol/kg)**	**Experimental CO_2 captured amount (mol/kg)**
C_2H_2-CO_2-C_2H_4 (1-1-98) 283 K	80.89	0.96	0.98
C_2H_2-CO_2-C_2H_4 (1-1-98) 298 K	64.42	0.78	0.84
C_2H_2-CO_2-C_2H_4 (1-1-98) 323 K	36.73	0.48	0.53
C_2H_2-CO_2-C_2H_4 (1-5-94) 298 K	21.37	0.60	1.52
C_2H_2-CO_2-C_2H_4 (1-9-90) 298 K (dry)	13.81	0.56	1.97
C_2H_2-CO_2-C_2H_4 (5-5-90) 298 K	11.04	2.65	0.55
C_2H_2-CO_2-C_2H_4 (1-9-90) 298 K (humid)	13.79	-	-

Supplementary Table 10. Experimental dynamic C_2H_4 productivity for different adsorbents.

	$C_2H_2/CO_2/C_2H_4$=1/9/90 (v/v/v) Flow rate: 5 mL/min					
	ZNU-6	NTU-67	Activated carbon	zeolite 5A	SIFSIX-2-Cu-i	SIFSIX-17-Ni
Mass (g)	0.58	1.20	0.98	1.74	0.52	0.82
Time 1[a] (min)	52.00	43.59	21.40	13.18	12.61	12.24
Time 2[b] (min)	196.00	84.38	34.19	45.89	170.08	83.82
Time 1 (min g^{-1})	89.56	36.17	21.84	7.58	24.20	14.96
Time 2 (min g^{-1})	337.58	70.03	34.89	26.40	326.44	102.42
Productivity per adsorption cycle (mol kg^{-1})	13.81	5.42	0.49	0.36	2.40	2.47
Productivity based on Time 1 (mol kg^{-1} h^{-1})	15.93	7.46	1.38	1.62	11.41	12.10
Productivity based on Time 2 (mol kg^{-1} h^{-1})	4.23	3.85	0.86	0.46	0.85	1.77

[a] Time 1 is the time when the second gas can be detected after C_2H_4;

[b] Time 2 is the time when C_A/C_0 reaches 1.0 for all the gases.

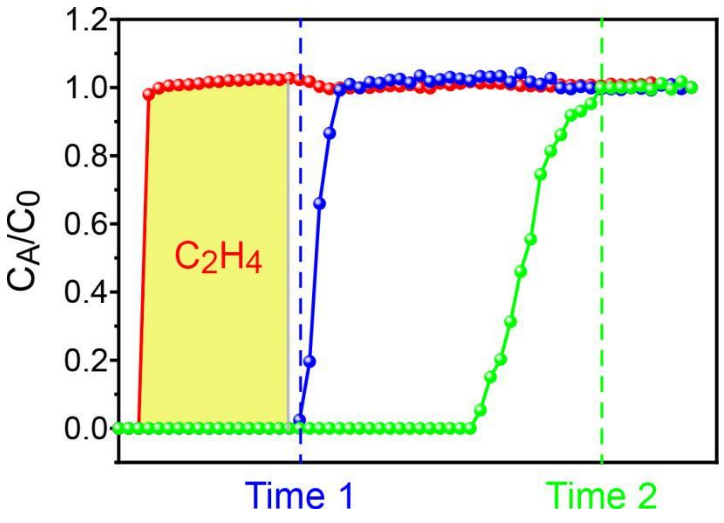

Supplementary Table 11. Experimental dynamic C_2H_4 productivity for ZNU-6 from different gas ratios and under different conditions.

	ZNU-6 Flow rate: 5 mL/min						
$C_2H_2/CO_2/C_2H_4$ (v/v/v)	1-1-98 283 K	1-1-98 298 K	1-1-98 323 K	1-5-94	1-9-90 dry	5-5-90	1-9-90 humid
Time 1 (min)	232.00	184.00	112.00	72.00	52.00	44.00	112.00
Time 2 (min)	284.00	248.00	180.00	192.00	196.00	180.00	180.00
Productivity per adsorption cycle (mol kg^{-1})	80.89	64.42	36.73	21.37	13.81	11.04	13.79
Productivity based on Time 1 (mol kg^{-1} h^{-1})	20.92	21.01	19.68	17.81	15.93	15.05	7.39
Productivity based on Time 2 (mol kg^{-1} h^{-1})	17.09	15.59	12.24	6.68	4.23	3.68	4.60

VI Supplementary References

[1] X.-H. Xiong, L. Zhang, W. Wang, N.-X. Zhu, L.-Z. Qin, H.-F. Huang, L.-L. Meng, Y.-Y. Xiong, M. Barboiu, D. Fenske, P. Hu, Z.-W. Wei, *ACS Appl. Mater. Interfaces* **2022**, *14*, 32105–32111.

[2] Q. Dong, Y. Huang, K. Hyeon-Deuk, I.-Y. Chang, J. Wan, C. Chen, J. Duan, W. Jin, S. Kitagawa, *Adv. Funct. Mater.* **2022**, 2203745.DOI: 10.1002/adfm.202203745.

[3] S. Mukherjee, N. Kumar, A. A. Bezrukov, K. Tan, T. Pham, K. A. Forrest, K. A. Oyekan, O. T. Qazvini, D. G. Madden, B. Space, M. J. Zaworotko, *Angew. Chem. Int. Ed.* **2021**, *60*, 10902–10909.

[4] X. Cui, K. Chen, H. Xing, Q. Yang, R. Krishna, Z. Bao, H. Wu, W. Zhou, X. Dong, Y. Han, B. Li, Q. Ren, M. J. Zaworotko, B. Chen, *Science* **2016**, *353*, 141–144.

[5] X. Cui, Q. Yang, L. Yang, R. Krishna, Z. Zhang, Z. Bao, H. Wu, Q. Ren, W. Zhou, B. Chen, H. Xing, *Adv. Mater.* **2017**, *29*, 1606929.

[6] O. Shekhah, Y. Belmabkhout, Z. Chen, V. Guillerm, A. Cairns, K. Adil, M. Eddaoudi, *Nat. Commun.* **2014**, *5*, 4228.

[7] R.-B. Lin, L. Li, H. Wu, H. Arman, B. Li, R.-G. Lin, W. Zhou, B. Chen, *J. Am. Chem. Soc.* **2017**, *139*, 8022–8028.

[8] Q. Dong, X. Zhang, S. Liu, R.-B. Lin, Y. Guo, Y. Ma, A. Yonezu, R. Krishna, G. Liu, J. Duan, R. Matsuda, W. Jin, B. Chen, *Angew. Chem. Int. Ed.* **2020**, *59*, 22756–22762.

[9] B. Li, X. Cui, D. O'Nolan, H.-M. Wen, M. Jiang, R. Krishna, H. Wu, R.-B. Lin, Y.-S. Chen, D. Yuan, H. Xing, W. Zhou, Q. Ren, G. Qian, M. J. Zaworotko, B. Chen, *Adv. Mater.* **2017**, *29*, 1704210.

[10] M. Jiang, B. Li, X. Cui, Q. Yang, Z. Bao, Y. Yang, H. Wu, W. Zhou, B. Chen, H. Xing, *ACS Appl. Mater. Interfaces* **2018**, *10*, 16628–16635.

[11] Z. Zhang, Q. Ding, J. Cui, H. Xing, *Small* **2020**, *16*, 2005360.

[12] D. O'Nolan, A. Kumar, K.-J. Chen, S. Mukherjee, D. G. Madden, M. J. Zaworotko, *ACS Appl. Nano Mater.* **2018**, *1*, 6000–6004.

[13] N. Xu, J. Hu, L. Wang, D. Luo, W. Sun, Y. Li, Y. Hu, D. Wang, X. Cui, H. Xing, Y. Zhang, *Chem. Eng. J.* **2022**, *450*, 138034.

[14] J. Wang, Y. Zhang, P. Zhang, J. Hu, R.-B. Lin, Q. Deng, Z. Zeng, H. Xing, S. Deng, B. Chen, *J. Am. Chem. Soc.* **2020**, *142*, 9744–9751.

[15] L. Wang, N. Xu, Y. Hu, W. Sun, R. Krishna, J. Li, Y. Jiang, S. Duttwyler, Y. Zhang, *Nano Res.* **2022** (in revision).

[16] H. Li, C. Liu, C. Chen, Z. Di, D. Yuan, J. Pang, W. Wei, M. Wu, M. Hong, *Angew. Chem. Int. Ed.* **2021**, *60*, 7547–7552.
